# Latitudinal patterns and environmental drivers of taxonomic, functional, and phylogenetic diversity of woody plants in western Amazonian *terra firme* forests

**DOI:** 10.3389/fpls.2022.978299

**Published:** 2022-10-07

**Authors:** Celina Ben Saadi, Luis Cayuela, Guillermo Bañares de Dios, Julia G. de Aledo, Laura Matas-Granados, Norma Salinas, María de los Ángeles La Torre Cuadros, Manuel J. Macía

**Affiliations:** ^1^ Departamento de Biología, Área de Botánica, Universidad Autónoma de Madrid, Madrid, Spain; ^2^ Departmento de Biología y Geología, Física y Química Inorgánica, Universidad Rey Juan Carlos, Móstoles, Spain; ^3^ Centro de Investigación en Biodiversidad y Cambio Global (CIBC-UAM), Universidad Autónoma de Madrid, Madrid, Spain; ^4^ Sección Quíımica, Pontificia Universidad Católica del Perú, Lima, Peru; ^5^ School of Geography and Environment, University of Oxford, Oxfordshire, United Kingdom; ^6^ Departamento de Ciencias Agrarias, Universidad Científica del Sur, Villa el Salvador, Peru; ^7^ Departamento de Manejo Forestal, Universidad Nacional Agraria La Molina, Lima, Peru

**Keywords:** latitudinal diversity gradient, taxonomic diversity (TD), functional diversity (FD), phylogenetic diversity (PD), environmental filtering, favourability hypothesis, tropical *terra firme* forest, woody plant

## Abstract

Elucidating how environmental factors drive plant species distributions and how they affect latitudinal diversity gradients, remain essential questions in ecology and biogeography. In this study we aimed: 1) to investigate the relationships between all three diversity attributes, *i.e*., taxonomic diversity (TD), functional diversity (FD), and phylogenetic diversity (PD); 2) to quantify the latitudinal variation in these diversity attributes in western Amazonian *terra firme* forests; and 3) to understand how climatic and edaphic drivers contribute to explaining diversity patterns. We inventoried *ca.* 15,000 individuals from *ca.* 1,250 species, and obtained functional trait records for *ca.* 5,000 woody plant individuals in 50 plots of 0.1 ha located in five *terra firme* forest sites spread over a latitudinal gradient of 1200 km covering *ca*. 10°C in latitude in western Amazonia. We calculated all three diversity attributes using Hill numbers: *q* = 0 (richness), *q* = 1 (richness weighted by relative abundance), and *q* = 2 (richness weighted by dominance). Generalized linear mixed models were constructed for each diversity attribute to test the effects of different uncorrelated environmental predictors comprising the temperature seasonality, annual precipitation, soil pH and soil bulk density, as well as accounting for the effect of spatial autocorrelation, *i.e*., plots aggregated within sites. We confirmed that TD (*q* = 0, *q* = 1, and *q* = 2), FD (*q* = 0, *q* = 1, and *q* = 2), and PD (*q* = 0) increased monotonically towards the Equator following the latitudinal diversity gradient. The importance of rare species could explain the lack of a pattern for PD (*q* = 1 and *q* = 2). Temperature seasonality, which was highly correlated with latitude, and annual precipitation were the main environmental drivers of variations in TD, FD, and PD. All three diversity attributes increased with lower temperature seasonality, higher annual precipitation, and lower soil pH. We confirmed the existence of latitudinal diversity gradients for TD, FD, and PD in hyperdiverse Amazonian *terra firme* forests. Our results agree well with the predictions of the environmental filtering principle and the favourability hypothesis, even acting in a 10°C latitudinal range within tropical climates.

## Introduction

Environmental drivers are key factors that determine plant diversity patterns at different spatial scales (McGill et al., 2006; HilleRisLambers et al., 2012; Arellano et al., 2016), but we still lack a comprehensive understanding of how environmental drivers act in hyperdiverse tropical forests (Wieczynski et al., 2019; Mori et al., 2021; Bañares de Dios et al., 2022). Diversity patterns have been traditionally explored in terms of taxonomic diversity (TD), *i.e*., species identity (Swenson, 2011; Swenson et al., 2012c). TD provides insights into the distribution of diversity and its underlying mechanisms (Gentry, 1982) but it does not consider two important diversity attributes: functional adaptations and evolutionary history (Swenson, 2011; Dawson et al., 2013; Chao et al., 2014; López et al., 2016). Functional diversity (FD) provides information about phenotypic adaptation to the environment and it is measured through functional traits (Cornelissen et al., 2003; Pérez-Harguindeguy et al., 2013; Córdova-Tapia & Zambrano, 2015). Due to the adaptive information provided, the functional perspective has increased in importance compared with the classic taxonomic approach for answering questions about the interactions between communities and their surrounding environment (Cornelissen et al., 2003; Violle et al., 2007; Swenson, 2012a; Poorter et al., 2017; Poorter et al., 2018). In addition, phylogenetic diversity (PD) is defined as the degree of phylogenetic relatedness among co-occurring species (López et al., 2016; Pellens & Grandcolas, 2016; Massante et al., 2019). The use of PD has grown in popularity in recent decades because the evolutionary history of communities provide insights into species relationships and functional trait evolution (Lean & Maclaurin, 2016; Pellens & Grandcolas, 2016).

Environmental drivers such as climate and edaphic properties contribute to explaining diversity patterns in tropical *terra firme* forests, *i.e.*, non-flooding. Water availability and soil fertility are abiotic factors with key effects on favouring plant growth, and thus are the main drivers of variations in spatial diversity in lowland tropical forests (Gentry, 1988; Fortunel et al., 2014; Honorio Coronado et al., 2015; Poorter et al., 2017). The available water depends mainly on temperature, precipitation, and soil texture (Sollins, 1998; Clarholm & Skyllberg, 2013; Moles et al., 2014). Crucially, tropical rainforests experience important changes in water availability throughout the year due to precipitation and temperature seasonality (Malhi & Wright, 2004; Moles et al., 2014; Malizia et al., 2020). Therefore, the main climatic constraint on plant growth and survival is usually the severity and duration of the dry season (Malhi & Wright, 2004; Aubry-Kientz et al., 2015; Honorio Coronado et al., 2015), even under tropical climates. This seasonality follows a latitudinal pattern where it intensifies towards higher latitudes. Accordingly, climatic seasonality is the main driver of the global latitudinal diversity gradient, which is one of the most widely accepted diversity patterns on Earth, where TD increases from the poles towards the Equator as a result of climatic favourability (Fischer, 1960; Gentry, 1982; Willig et al., 2003; Weiser et al., 2007; Swenson et al., 2012c; Malizia et al., 2020). FD has also been shown to increase with decreasing latitude (Swenson et al., 2012b; Wieczynski et al., 2019), where the number of functional strategies increases as the climatic conditions become more benign (Fischer, 1960; Swenson et al., 2012b; Moles et al., 2014; Wieczynski et al., 2019). In woody angiosperms, PD also increases towards the Equator (Qian et al., 2013; Kerkhoff et al., 2014; Qian et al., 2017; Massante et al., 2019), partly due to the favourable climatic conditions found at lower latitudes.

Another important aspect of water availability is the soil water content, which depends greatly on the soil texture because it controls aeration, drainage and humidity retention capacity (Sollins, 1998; Rodrigues et al., 2019; Hofhansl et al., 2020). In addition, variability in soil fertility directly affects plant diversity (Sanford & Cuevas, 1996; Garibaldi et al., 2014; Bañares de Dios et al., 2022). Most *terra firme* tropical soils are oligotrophic but they are rich in nitrogen (Sollins, 1998) with quite acidic pH values (Herrera et al., 1978; Medina & Cuevas, 1989; Sollins, 1998). The fertility of tropical soils depends on pH because it determines the availability of base-metal cations and toxicity of aluminum (Herrera et al., 1978; Sollins, 1998; Nelson & Su, 2010; Bañares de Dios et al., 2022). Nevertheless, species seem to tolerate these low nutrient, acidic, and high aluminum conditions (Herrera et al., 1978; Medina & Cuevas, 1989; Mori et al., 2021). Hence, both the soil nutrient availability and water content play critical roles in environmental filtering (Katabuchi et al., 2012; Rodrigues et al., 2019; Hofhansl et al., 2020), which is mainly dependent on the soil pH and texture (Sollins, 1998; Nelson & Su, 2010). In addition, the soil organic matter content favours moisture retention, cation exchange and nutrient turnover, thereby affecting the soil texture and fertility and thus improving soil quality (Craswell & Lefroy, 2001; Athira et al., 2019; Bañares de Dios et al., 2022). The bulk density is determined by the soil texture and it has an inverse relationship with the soil organic matter content, so it can be used as a proxy for the soil quality (Bauer, 1974; Athira et al., 2019; de la Cruz-Amo et al., 2020).

The main goal of the present study was to investigate the patterns of woody plant diversity in western Amazonian *terra firme* forests along a 1,200 km latitudinal gradient. Amazonian rainforests, specifically western Amazonian forests, harbour among the most hyperdiverse floras worldwide (Gentry, 1988; Wright, 2002; Honorio Coronado et al., 2015; Brooks, 2018). Amazonian rainforests are characterized by high abundance and diversity of woody angiosperms, where a limited number of species dominate the community (Pitman et al., 2013; Draper et al., 2021). The relatively low number of dominant taxa means that tropical communities usually harbour extremely large numbers of rare species (Ter Steege et al., 2013; Leitão et al., 2016; Draper et al., 2021; Cazzolla Gatti et al., 2022). Many studies have been conducted in Amazonian rainforests over the last century, but none analysed the latitudinal gradients of woody plants within tropical regions by considering all three diversity attributes comprising TD, FD, and PD. In this study we specifically aimed: 1) to investigate the relationship between all three diversity attributes; 2) to quantify the latitudinal variations in TD, FD and PD; and 3) to understand how climatic and edaphic drivers contribute to explaining diversity patterns. We hypothesized that all three diversity attributes would increase towards equatorial latitudes, mainly due to a reduction in seasonality harshness, with differential contributions of climatic drivers and soil properties, in agreement with environmental filtering.

## Materials and methods

### Study area and taxonomic characterization of plant communities

This study was conducted in western Amazonian forests which comprise several forest types, but the most extended are *terra firme* forests (Herrera et al., 1978). *Terra firme* forests are highly diverse and can contain up to 300 tree species per hectare (Gentry, 1988; Valencia et al., 1994). Western Amazonian forests formed on Pliocene and Pleistocene sediments from the Andes, which are fairly young and fertile soils compared with those in central and eastern Amazonia (Pitman et al., 2001; Hoorn et al., 2010; Lamarre et al., 2012; Honorio Coronado et al., 2015; Nobre et al., 2019). These forests have low annual thermal variability, high precipitation, and relatively well-drained fertile soils (Gentry, 1988; Burnham & Johnson, 2004; Brooks, 2018).

**Figure 1:**
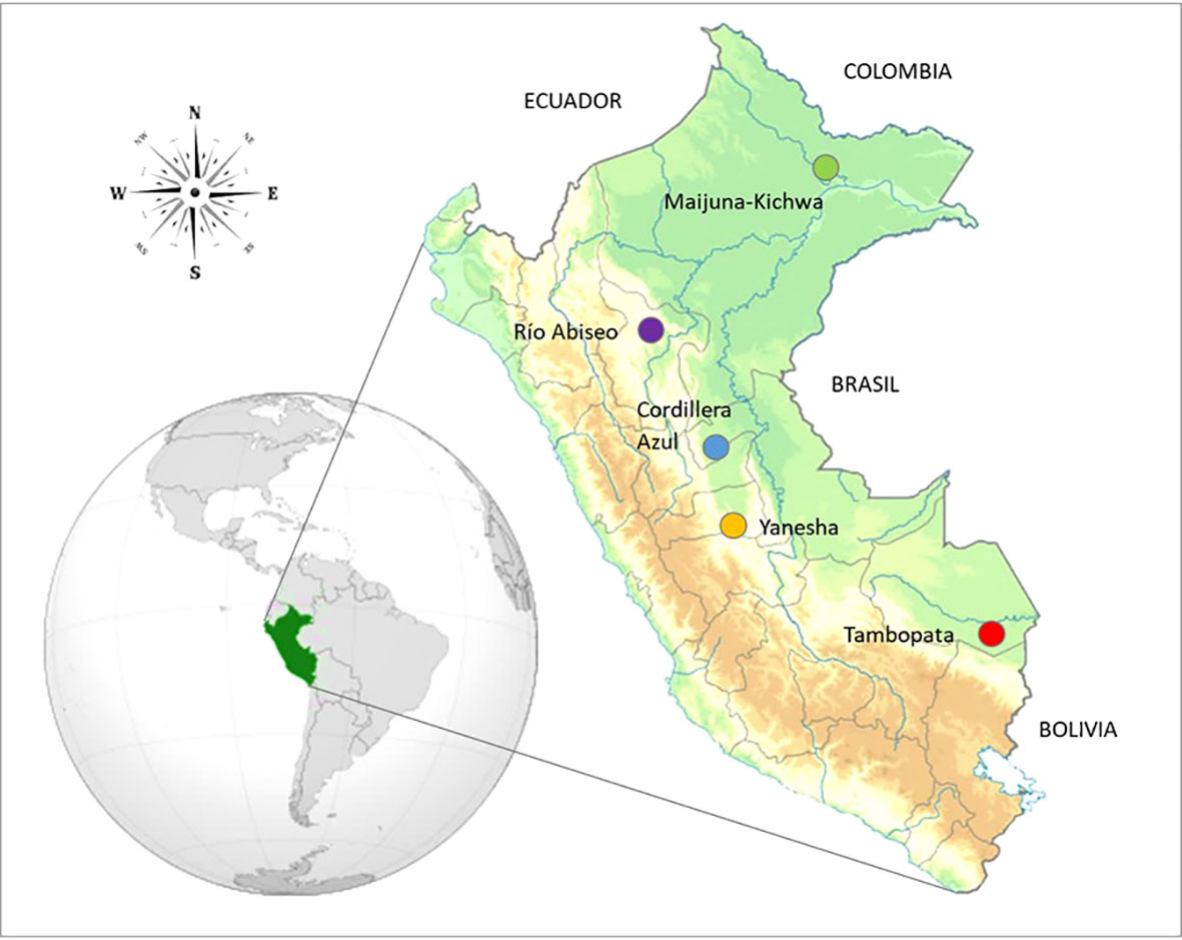
Map of Peru with coloured dots indicating the five regions studied in Western Amazonia. Top to bottom: Maijuna-Kichwa Regional Conservation Area, Río Abiseo National Park, Buffer Zone of Cordillera Azul National Park, Yanesha Comunal Reserve and Tambopata National Reserve.

The study area encompassed 50 plots sampled throughout five western Amazonian regions in Peru spanning a 1,200 km latitudinal gradient from –2.9496°C to –12.8270°C and covering *ca*. 10°C in latitude (**Figure 1**, **Table S1**). The study regions corresponded to the protected areas of Tambopata National Reserve (Madre de Dios), Buffer Zone of Yanesha Communal Reserve (Pasco), Buffer Zone of Cordillera Azul National Park (Ucayali), Río Abiseo National Park (San Martín), and Maijuna-Kichwa Regional Conservation Area (Loreto). In each region 10 plots of 0.1 ha (50 × 20 m) were established according to Arellano et al. (2016). Plots were located in areas of well conserved *terra firme* lowland and submontane forests according to the biome classification of Britto (2017), and they were established at least 300 m apart from each other to minimize spatial autocorrelation. All woody plant individuals, including trees, lianas, and hemiepiphytes, rooting inside the plot limits with a diameter at breast height (DBH) ≥ 2.5 cm were included. Plot based studies usually apply an inclusion criterion of DBH ≥ 10 cm, so our study included a high number of juvenile and understory individuals that are rarely considered (Gentry, 1982; Arellano et al., 2016; Draper et al., 2021), which could increase the TD and PD by including typically smaller species, as well as FD by considering more understory individuals that grow in shadier conditions (Niinemets, 2010; Weerasinghe et al., 2014). Up to three individuals from each taxon were sampled in each region to obtain herbarium sheet duplicates and replicates to measure functional traits. Collected vouchers specimens were identified and stored primarily in the USM herbarium, with some duplicates at MOL herbarium. The herbarium acronyms followed Thiers (2021). Taxonomic names were standardized using the ‘Taxostand’ package in R (Cayuela et al., 2012).

### Climatic and edaphic characterisation of study sites

Climatic data for each plot were retrieved from the CHELSA database (Karger et al., 2019). All 19 CHELSA temperature and precipitation related bioclimatic variables were considered (**Table S2**).

Five soil subsamples were collected in a zigzag pattern within the plot limits and mixed to obtain one representative sample for each plot. A metallic cylinder was used to extract subsamples of the first 15 cm of the soil layer below plant debris. After mixing and air drying, the soil samples were sieved through a 2 mm mesh to separate the organic fraction comprising organic matter debris, such as leaves and roots, and the coarse fraction. The remaining soil corresponded to the fine fraction which was used for chemical and textural analyses (**Table S3**). The pH was measured in distilled water as the real acidity and in KCl as the potential acidity. The organic carbon content and total N, S, and C contents were measured using the LECO/Dumas direct combustion method. The available Al contents and those of macronutrients (P, Ca, Mg, Na, and K) and micronutrients (Fe, Co, Cu, Mn, Ni, and Zn) were measured by extraction with the Melich III method followed by an inductively coupled plasma mass spectrometry. Textural analyses were performed using the hydrometer method after adding dispersal solution. Soil mineralogy and clay contents were measured by X-ray diffraction. The soil sampling and characterization analyses were based on the protocols described by Arellano et al. (2016).

To avoid collinearity, we calculated Pearson’s correlation coefficients *(r)* between all environmental variables (**Figure S1**), *i.e*., climate, soil, and latitude. Most of the environmental variables were highly correlated, so we selected two climatic and two edaphic uncorrelated variables based on previous studies. The selected climatic factors comprised the temperature seasonality (°C) and annual precipitation (mm/year) because they reflect climatic limitations on plant growth (Malhi & Wright, 2004; Aubry-Kientz et al., 2015). The selected edaphic factors comprised the pH as an approximation of soil fertility (Sollins, 1998; Nelson & Su, 2010) and the soil bulk density (g/cm^3^) to consider the soil texture and organic matter content (Bauer, 1974; Motavalli et al., 1995; Athira et al., 2019).

### Functional characterization of plant communities

Three functional traits comprising the specific leaf area (SLA), leaf thickness (LT) and wood density (WD) were measured for each individual collected. These traits are used extensively because they are robust indicators of the functional strategies of woody plants in the leaf (Wright et al., 2004) and wood economy spectrum (Chave et al., 2009). Five mature leaves were used to measure the foliar traits. SLA (mm^2^/mg) was calculated as the ratio between the leaf surface area measured with a CI-202 Portable Laser Leaf Area Meter (CID Bio-Science, WA, USA) and dry mass after drying for 48 h at 80°CC. SLA measurements included all of the leaf structures. LT (mm) was measured with a digital calliper. The branch wood density was used as an approximation for WD (Swenson & Enquist, 2008). For each individual collected, a 10 cm branch section was peeled to remove the bark and its dimensions were measured with a digital calliper to calculate the fresh volume. WD (g/cm^3^) was calculated as the ratio between the dry mass after drying for 48 h at 80°CC and the fresh volume. All measurements and calculations followed standardized protocols (Cornelissen et al., 2003). Only individuals with records for all three functional traits were included for functional characterization. Mean trait values were calculated for each taxon.

### Phylogenetic characterization of plant communities

We obtained a phylogenetic tree using the *V.PhyloMaker* package in R (Jin & Qian, 2019). This package allowed us to prune a pre-existing mega-tree of vascular plants based on the phylogenies of Smith and Brown (2018) and Zanne et al. (2014) for seed plants and pteridophytes, respectively, with a given list of species. The ‘phylo.maker’ function was used with the arguments *nodes=nodes.info.1* and *scenarios=S3*.

### Diversity measurements

We calculated Hill numbers for TD, FD and PD. Hill numbers only differ in terms of the parameter *q*, which determines the sensitivity to relative abundances (Hill, 1973). For TD (Chao et al., 2014): *q* = 0 denotes the species richness (*i.e*., number of species), *q* = 1 represents the richness considering relative abundance (*i.e*., Shannon’s diversity), and *q* = 2 is the richness considering dominance (*i.e*., inverse of Simpson’s index). Hence, the contribution of rare species to diversity decreases as the value of the parameter *q* increases. Hill numbers were typically only used for TD but Chao et al. (2010) extended them to PD based on the phylogenetic distances between species, and Chiu and Chao (2014) extended them to FD based on the functional distances between species traits.

By considering the Hill numbers for all three diversity attributes we can obtain a unified framework of attribute diversity, where each component is measured in different units or entities (Chao et al., 2014). TD is measured as the effective number of taxonomic entities, so the attribute value is unity for each taxon. For FD, the attribute value is the functional distance between each pair of taxa based on functional traits, and thus it is measured as the effective number of functional entities. PD is measured as the effective number of phylogenetic entities, where the attribute value is the length of each branch segment. All entities are treated as taxonomically, functionally and phylogenetically equally distinct. Hill numbers for *q* = 0, *q* = 1, and *q* = 2 were obtained for the three diversity attributes using the ‘renyi’ function in the *vegan* package for TD, with the code provided by Chiu and Chao (2014) for FD, and with the ‘ChaoPD’ function in the *entropart* package (Chao et al., 2010) for PD. Using different Hill numbers to explore diversity allowed us to examine how rare, abundant, and dominant species responded to the environmental factors and latitude.

### Data analyses

First, we explored the relationships between different Hill numbers as well as between Hill numbers and latitude using negative binomial generalized linear models (GLMs). To fit these models, we used the ‘glm.nb’ function in the *MASS* package (Venables & Ripley, 2002). To account for the variance explained by the models, we calculated Cragg and Uhler’s pseudo-R-squared (*pR^2^*) using the ‘pR2’ function in the *pscl* package (Jackman, 2020). We then constructed negative binomial generalized linear mixed models (GLMMs) to assess the effects of climatic and edaphic variables on the Hill numbers for each diversity attribute by using the ‘glmer.nb’ function in the *lme4* package (Bates et al., 2015). Environmental variables comprising the temperature seasonality, annual precipitation, soil pH and soil bulk density were included as fixed effects, whereas region (n = 5) was treated as a random factor to account for potential spatial autocorrelation among plots sampled within the same region. The latitude was not a factor itself but instead it was a surrogate for climatic gradients (Willig et al., 2003), so it was not included in the models. Nevertheless, it was indirectly considered because it was significantly negatively correlated with temperature seasonality. Temperature and seasonality have been shown to reflect the effects of latitudinal variation (Willig et al., 2003; Swenson et al., 2012b), thereby justifying our approach of using temperature seasonality as an accurate proxy for latitude in the models. Interaction terms between predictors were not included. Model selection was conducted based on Akaike’s Information Criterion corrected for small sample size (AICc). When models had a difference in AICc ≥ 2, the model with the highest AICc was considered less than optimal and rejected. When two or more models had a difference in AICc< 2, we selected the most complex to generate model predictions. We calculated two components of the pseudo-R-squared for GLMMs: marginal ( ${\text{R}}_{m}^{2}$ ) and conditional ( ${\text{R}}_{c}^{2}$ ) coefficients, which represent the variance explained by fixed effects and by both random and fixed effects, respectively (Nakagawa & Schielzeth, 2013). We used the ‘r.squaredGLMM’ function in the *MuMIn* R package (Barton, 2013). All analyses were conducted in R v4.2.1 (R Core Team, 2021).

## Results

### Environmental and taxonomic overview

The temperature seasonality ranged from 4.16°C in Maijuna to 9.30°C in Tambopata (**Table S1**) and it had a strong significant correlation with latitude (*r* = –0.97; **Table S4**). The maximum annual precipitation was recorded at Cordillera Azul with 3948 mm and the minimum at Río Abiseo with 1756 mm (**Table S1**), and it had no significant correlation with latitude (*r* = –0.09; **Table S4**). Among the edaphic properties, the soil bulk density and pH were not correlated with each other (*r* = –0.26; **Table S4**). Both variables were weakly correlated with the annual precipitation (bulk density: *r* = 0.40; pH: *r* = –0.29; **Table S4**), but not with the temperature seasonality (bulk density: *r* = 0.25; pH: *r* = –0.18; **Table S4**) or latitude (bulk density: *r* = –0.20; pH: *r* = 0.11; **Table S4**). The bulk density ranged from 0.61 to 1.00 g/cm^3^ and it varied fairly heterogeneously among the sites. The pH ranged from 3.78 to 5.46 (**Table S1**), although all of the pH values above 5.0 were determined in white sand soils in seven plots in Río Abiseo.

Overall, 14,681 individuals were inventoried in five regions belonging to 2,211 species (See **Table S5** for the list of species). A total of 302 taxa were identified in Tambopata, 467 taxa in Yanesha, 511 taxa in Cordillera Azul, 154 taxa in Río Abiseo and 542 taxa in Maijuna (**Table S6**). Among the functional data, 274 taxa had records for all functional traits in Tambopata, 442 taxa in Yanesha, 466 taxa in Cordillera Azul, 269 taxa in Río Abiseo and 450 taxa in Maijuna (**Table S6**). The generated phylogenetic tree included 302 taxa present in Tambopata, 467 taxa in Yanesha, 511 taxa in Cordillera Azul, 292 taxa in Río Abiseo and 542 taxa in Maijuna (**Table S6**).

### Correlation between diversity attributes

**Figure 2:**
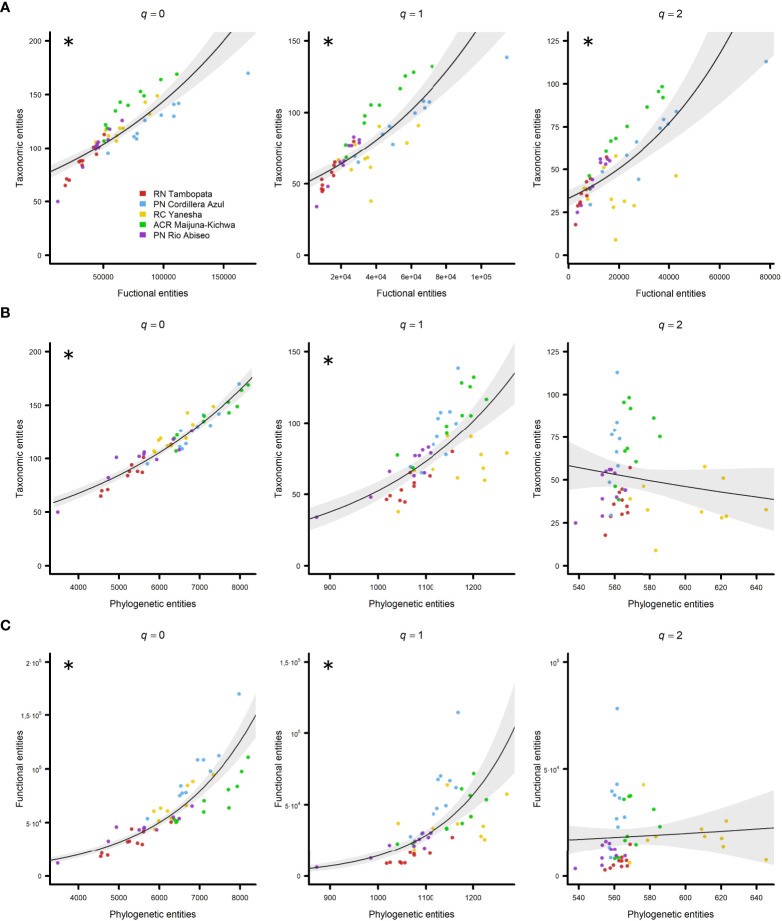
Relationship between diversity attributes in all five regions studied in Western Amazonia represented by negative binomial generalized linear models (GLMs) with 95% confidence intervals. **(A)** Taxonomic vs. functional entities, **(B)** taxonomic vs. phylogenetic entities, and **(C)** functional vs. phylogenetic entities. Hill numbers were considered as diversity indexes: q = 0 (left column), q = 1 (central column), and q = 2 (right column). Regions are represented by different colours (see the legend). Significant pseudo R-squared (*pR^2^*) *v*alues are marked with asterisks.

When all sites were considered together in the analysis, TD and FD were positively and significantly correlated with each other for all three Hill numbers *q* = 0 (*pR^2^* = 0.71), *q* = 1 (*pR^2^* = 0.66), and *q* = 2 (*pR^2^* = 0.50) (**Figure 2A**; **Table 1**). However, when PD was considered with either TD or FD, the only Hill numbers with positive and significant correlations were *q* = 0 (with TD: *pR^2^* = 0.90; with FD: *pR^2^* = 0.81) and *q* = 1 (with TD: *pR^2^* = 0.51; with FD: *pR^2^* = 0.53) (**Figures 2B, C**; **Table 1**).

**Table 1:** Pseudo R-squared (*pR^2^*) values for negative binomial generalized linear models (GLMs) between diversity attributes for different Hill numbers: taxonomic diversity (TD), functional diversity (FD), and phylogenetic diversity (PD).

Study areas	Hill numbers	TD vs. FD	TD vs. PD	FD vs. PD
**All sites**	*q = 0*	0.71***	0.90***	0.81***
	*q = 1*	0.66***	0.51***	0.53***
	*q = 2*	0.50***	0.02	0.00
**Tambopata**	*q = 0*	0.81***	0.80***	0.78***
	*q = 1*	0.75***	0.70***	0.85***
	*q = 2*	0.76***	0.47**	0.62***
**Cordillera Azul**	*q = 0*	0.84***	0.84***	0.93***
	*q = 1*	0.87***	0.64***	0.77***
	*q = 2*	0.78***	0.03	0.13
**Yanesha**	*q = 0*	0.71***	0.67***	0.76***
	*q = 1*	0.21	0.26	0.07
	*q = 2*	0.01	0.00	0.07
**Maijuna**	*q = 0*	0.79***	0.82***	0.86***
	*q = 1*	0.86***	0.76***	0.75***
	*q = 2*	0.90***	0.13	0.10
**Río Abiseo**	*q = 0*	0.89***	0.83***	0.78***
	*q = 1*	0.88***	0.85***	0.88***
	*q = 2*	0.83***	0.33*	0.29*

Significance levels represented by: (*) when p<0.05, (**) when p<0.01 and (***) when p<0.001.

### Latitudinal patterns

**Figure 3:**
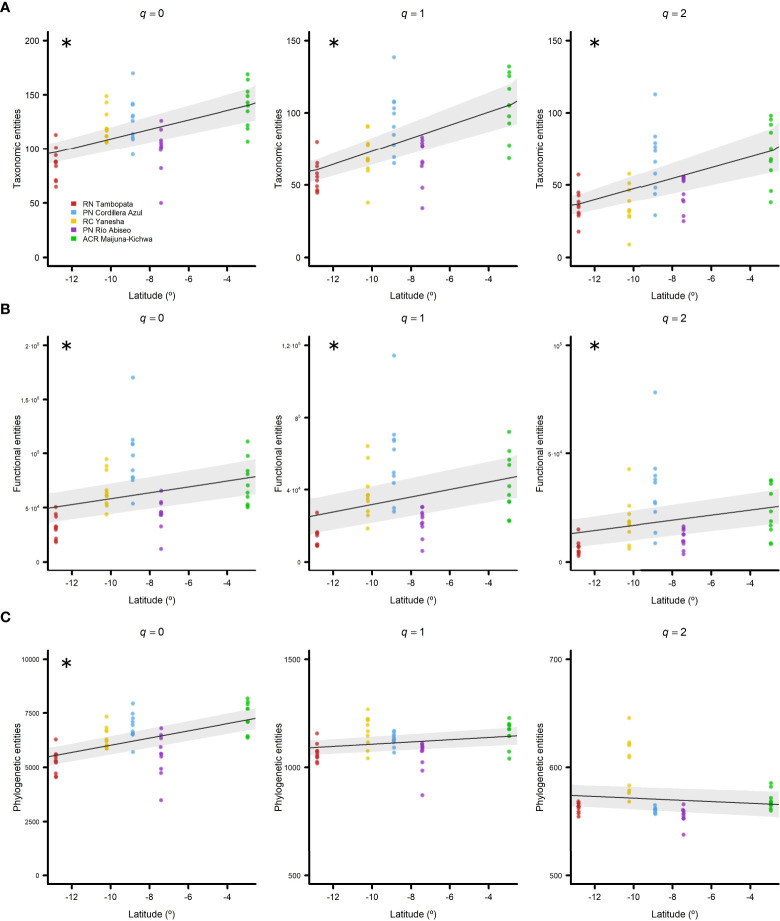
Diversity latitudinal trends for all five regions studied in Western Amazonia represented by negative binomial generalized linear models (GLMs) with 95% confidence intervals. **(A)** Taxonomic, **(B)** functional, and **(C)** phylogenetic entities found against latitude in decimal degrees. Hill numbers were considered as diversity indexes: *q* = 0 (left column), *q* = 1 (central column), and *q* = 2 (right column). Regions are represented by different colours (see the legend). Significant pseudo R-squared (*pR^2^*) *v*alues are marked with asterisks.

When the TD patterns were explored across latitude, significant positive relationships were found for all three Hill numbers: *q* = 0 (*pR^2^* = 0.27), *q* = 1 (*pR^2^* = 0.34), and *q* = 2 (*pR^2^* = 0.28) (**Figure 3A**; **Table 2**), thereby indicating that diversity increases towards the Equator. For FD, all of the Hill numbers exhibited significant positive trends, but latitude explained a lower proportion of the variability in FD: *q* = 0 (*pR^2^* = 0.10), *q* = 1 (*pR^2^* = 0.12), and *q* = 2 (*pR^2^* = 0.10) (**Figure 3B**; **Table 2**). The only Hill number for PD that had a significant positive latitudinal trend was *q* = 0 (*pR^2^* = 0.27) (**Figure 3C**; **Table 2**).

**Table 2:** Pseudo R-squared (*pR^2^*) values for negative binomial generalized linear models (GLMs) between latitude and diversity attributes for different Hill numbers: taxonomic diversity (TD), functional diversity (FD) and phylogenetic diversity (PD).

Diversity atributes	Hill numbers	*pR^2^*
**TD**	*q = 0*	0.27***
	*q = 1*	0.34***
	*q = 2*	0.28***
**FD**	*q = 0*	0.10*
	*q = 1*	0.12**
	*q = 2*	0.10**
**PD**	*q = 0*	0.27***
	*q = 1*	0.05
	*q = 2*	0.01

Significance levels represented by: (*) when p<0.05, (**) when p<0.01 and (***) when p<0.001.

### Effects of environmental variables on TD, FD and PD

**Figure 4:**
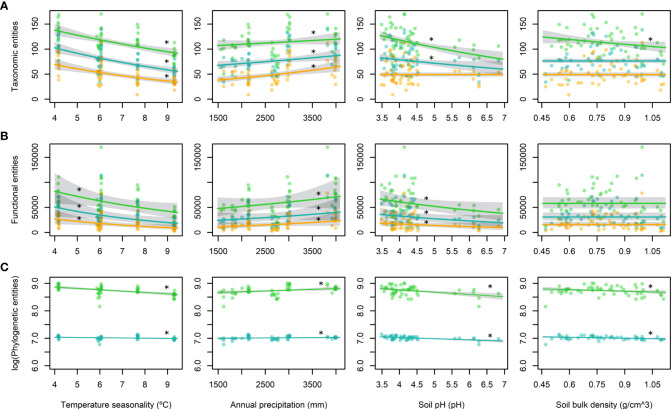
Predictions of the best-fit generalized linear mixed models (GLMMs) with 95% confidence intervals for each diversity attribute: **(A)** taxonomic entities, **(B)** functional entities, and **(C)** phylogenetic entities. Hill numbers are shown in different colours: *q* = 0 in green, *q* = 1 in blue, and *q* = 2 in yellow. Each column corresponds to one of the terms included in the models: temperature seasonality (left column), annual precipitation (central left column), soil pH (central right column), and soil bulk density (right column). Terms included in the best-fit model are marked with asterisks.

Our results indicated monotonic increases in all diversity attributes towards lower latitudes, except when we considered relative abundances (*q* = 1) and dominance (*q* = 2) for PD (**Figure 3B**). Analysis of the contributions of environmental variables to diversity indicated that climatic factors seemed to be responsible for the latitudinal diversity pattern, whereas the edaphic properties appeared to have different effects on common and rare species (**Figure 4**). The effect of temperature seasonality was significant for all diversity attributes irrespective of whether relative abundances and dominance were considered (**Figure 4**).

The best-fit model was selected for each diversity attribute and each Hill number based on the AICc values. All of the best-fit models for TD, FD, and PD included temperature seasonality, which was highly correlated with latitude (*r* = –0.97; **Table S4**), and annual precipitation as fixed terms (**Figure 4**; **Table 3**). Both the soil pH and density appeared in the best-fit models for TD with *q* = 0, and PD with *q* = 0 and *q* = 1 (**Figure 4**; **Table 3**). In addition, pH was also present in TD with *q* = 1 and all three best-fit models for FD (**Figure 4**; **Table 3**). The best-fit model for PD with *q* = 2 (**Table 3**) had no explanatory variables except for the random factor, *i.e.*, null model.

**Table 3:** Comparison of the best alternative models for Hill numbers of taxonomic diversity (TD), functional diversity (FD), and phylogenetic diversity (PD) as functions of temperature seasonality, annual precipitation, soil pH, and soil bulk density.

Diversity attribute	Hill numbers	Formulae for the best models	df	AICc	${\text{R}}_{m}^{2}$	${\text{R}}_{c}^{2}$
**TD**	q = 0	TD.q0 ~ (1|Region) + Temperature seasonality + Soil pH + Soil density	8	446.90	0.540	0.540
		**TD.q0 ~ (1|Region) + Temperature seasonality + Annual precipitation + Soil pH + Soil density**	**9**	**448.11**	**0.555**	**0.555**
	q = 1	TD.q1 ~ (1|Region) + Temperature seasonality + Soil pH	7	433.66	0.507	0.512
		**TD.q1 ~ (1|Region) + Temperature seasonality + Annual precipitation + Soil pH**	**8**	**433.17**	**0.556**	**0.556**
	q = 2	**TD.q2 ~ (1|Region) + Temperature seasonality + Annual precipitation**	**7**	**426.32**	**0.482**	**0.482**
**FD**	q = 0	FD.q0 ~ (1|Region) + Soil pH	6	1140.76	0.066	0.596
		FD.q0 ~ (1|Region) + Temperature seasonality + Soil pH	7	1139.81	0.329	0.583
		**FD.q0 ~ (1|Region) + Temperature seasonality + Annual precipitation + Soil pH**	**8**	**1141.35**	**0.415**	**0.584**
	q = 1	FD.q1 ~ (1|Region) + Soil pH	6	1098.98	0.045	0.623
		FD.q1 ~ (1|Region) + Temperature seasonality + Soil pH	7	1097.58	0.362	0.612
		**FD.q1 ~ (1|Region) + Temperature seasonality + Annual precipitation + Soil pH**	**8**	**1099.14**	**0.438**	**0.616**
	q = 2	FD.q2 ~ (1|Region) + Soil pH	6	1055.47	0.035	0.533
		FD.q2 ~ (1|Region) + Temperature seasonality + Annual precipitation	7	1054.37	0.378	0.538
		**FD.q2 ~ (1|Region) + Temperature seasonality + Annual precipitation + Soil pH**	**8**	**1055.04**	**0.407**	**0.535**
**PD**	q = 0	PD.q0 ~ (1|Region) + Temperature seasonality + Annual precipitation + Soil pH	8	812.56	0.533	0.533
		**PD.q0 ~ (1|Region) + Temperature seasonality + Annual precipitation + Soil pH + Soil density**	**9**	**812.27**	**0.560**	**0.560**
	q = 1	PD.q1 ~ (1|Region) + Soil pH	6	563.00	0.141	0.319
		PD.q1 ~ (1|Region) + Temperature seasonality + Soil pH	7	562.85	0.276	0.323
		PD.q1 ~ (1|Region) + Temperature seasonality + Soil pH + Soil density	**8**	561.65	0.377	0.377
		**PD.q1 ~ (1|Region) + Temperature seasonality + Annual precipitation + Soil pH + Soil density**	**9**	**563.48**	**0.388**	**0.388**
	q = 2	**PD.q2 ~ (1|Region)**	**5**	**440.80**	**0.000**	**0.290**
		PD.q2 ~ (1|Region) + Soil pH	6	442.12	0.033	0.250
		PD.q2 ~ (1|Region) + Soil density	6	442.44	0.023	0.260
		PD.q2 ~ (1|Region) + Soil pH + Soil density	7	442.12	0.200	0.229

Region is included as a random factor. df , degrees of freedom; AICc , Akaike’s Information Criterion corrected for small sample sizes; ${\text{R}}_{m}^{2}$ , pseudo-R-squared accounting for fixed effects; ${\text{R}}_{c}^{2}$ , pseudo-R-squared accounting for both random and fixed effects. The best-fit models are highlighted in bold. Significant terms for the best-fit models are underlined.

## Discussion

### Correlations between diversity attributes

The relationship between TD and FD is clear because, a larger potential range of functional strategies can be found when more species are present (Kooyman et al., 2012). We found that TD and FD were positively and significantly correlated (**Figure 2A**) even when more weight was given to abundant and dominant taxa (*q* = 1 and especially *q* = 2), thereby suggesting that although dominant species exhibited a narrower spectrum of functional strategies compared with rare species, they were not limited to the same suite of functional strategies, and thus FD increased as the number of dominant species increased (**Figure 2A**, *q* = 2). The relationship between TD and FD was in agreement with previous studies conducted at a global scale (Li et al., 2018), in the New World (Swenson et al., 2012b; Lamanna et al., 2014), and North America (Swenson & Weiser, 2014).

In addition, TD is closely related to PD, and thus they should be strongly positively correlated (Honorio Coronado et al., 2015). However, local speciation or extinction events could lead to the adaptive radiation of a few lineages and the opposite trend, *i.e*., high TD and low PD (Losos, 2008; Massante et al., 2019). Our results showed that TD and PD increased together (**Figure 2B**, *q* = 0 and *q* = 1), so there seemed to be no indication of high diversification by a few lineages as shown in a previous study (Giehl & Jarenkow, 2012). However, this was not the case when we considered dominance (**Figure 2B**, *q* = 2) because a few clades appeared to account for many of the dominant species (Draper et al., 2021).

Finally, phylogenetic relatedness involves some degree of evolutionary preservation of the adaptive strategies reflected in functional traits (Swenson et al., 2012c; López et al., 2016; Coelho de Souza et al., 2019). Thus, if FD is strongly influenced by a shared evolutionary history, there should be a positive correlation between phylogenetic proximity and ecological similarity (Webb, 2000; Webb et al., 2008; Kooyman et al., 2012). However, not all functional traits are necessarily phylogenetically conserved (Cavender-Bares et al., 2004; Swenson & Enquist, 2009; López et al., 2016), and strong adaptation of related lineages to a heterogeneous environment leads to high FD and low PD, thereby inverting the trend (Losos, 2008; López et al., 2016). The positive relationship between FD and PD (**Figure 2C**, *q* = 0 and *q* = 1) suggested that the functional traits in our study were phylogenetically conserved. However, when dominance was considered (*q* = 2), the dominant clades probably had a limited set of functional adaptations and/or these traits were not phylogenetically conserved. The absence of significant relationships between TD–PD and FD–PD when considering dominance (**Figures 2B, C**, *q* = 2) were probably due to seven plots in Yanesha with surprisingly high PD but relatively low TD, *i.e*., a few species from distant lineages dominated the community, and FD, *i.e*., a few functional strategies that appeared in distant lineages dominated the community.

Regardless of the causes, the positive relationships between the three diversity attributes showed that if we take action to preserve and conserve communities with high TD, we are also indirectly preserving FD and PD, at least in western Amazonian *terra firme* forests.

### Latitudinal patterns

Overall, our results indicated that the three diversity attributes increased monotonically towards the Equator in western Amazonian *terra firme* forests (**Figure 3**), where they consistently followed the latitudinal diversity gradient. These findings agree well with the predictions of the environmental filtering principle, which states that communities are shaped by abiotic deterministic factors (Götzenberger et al., 2012; López et al., 2016; Poorter et al., 2017), and the favourability hypothesis, which maintains that the effect of environmental filtering becomes more restrictive when the environmental conditions are less favourable (Swenson et al., 2012b; Lamanna et al., 2014; Wieczynski et al., 2019), and thus they forecast that TD, FD, and PD will increase towards lower latitudes due to climatic favourability (Fischer, 1960; Weiser et al., 2007; Swenson et al., 2012c; Qian et al., 2013; Qian et al., 2017; Kubota et al., 2018; Massante et al., 2019; Wieczynski et al., 2019). In addition, our results showed that the latitudinal decrease in all three diversity attributes was noticeable within tropical areas, even in a 10°C latitudinal gradient. Thus, although tropical environments have benign climatic conditions in global terms, environmental filtering and climatic favourability are still important for these species-rich communities (Fortunel et al., 2014). In fact, several studies found that environmental filtering had a stronger effect in species-rich communities, such as tropical forests, compared with species-poor communities (Lamanna et al., 2014; Swenson & Weiser, 2014; Li et al., 2018)

We found a monotonic increase in TD with decreasing latitude, which was consistent for all Hill numbers (**Figure 3A**), thereby indicating that this pattern remained regardless of whether the relative abundances (*q* = 1) and dominance (*q* = 2) of taxa were considered. Our results showed that TD increased towards the Equator, which is a widely accepted trend (Fischer, 1960; Gentry, 1982; Willig et al., 2003; Weiser et al., 2007). Some recent studies of latitudinal gradients obtained similar results in terms of the species richness in North America (Qian et al., 2013) and South America (Cazzolla Gatti et al., 2022).

Our results indicated a monotonic increase in FD with decreasing latitude (**Figure 3B**), which is in agreement with previous studies of woody plants in the New World (Swenson et al., 2012b; Lamanna et al., 2014). This finding was consistent for all Hill numbers, thereby indicating that this pattern remained regardless of whether relative abundances (*q* = 1) and dominance (*q* = 2) were considered. The reduction in functional entities when abundance and dominance were considered could be explained by the absence of rare species with rare ecological strategies, *i.e*., uncommon combinations of functional traits, which were greatly responsible for the FD of the community (Mouillot et al., 2013; Leitão et al., 2016).

Finally, we found a monotonic increase in PD of woody plants with decreasing latitude only for the Hill number of *q* = 0 (**Figure 3C**). This pattern is consistent with recent studies of woody angiosperms in the New World (Kerkhoff et al., 2014), North America (Qian et al., 2013), and even at a global scale (Qian et al., 2017). The results obtained by Massante et al. (2019) appear to contradict ours because they found a worldwide increase in PD of woody plants towards higher latitudes. However, they also considered gymnosperms in their analyses, which usually have greater tolerance of harsher conditions at high latitudes, and the trend inverted when they were excluded. In addition, no significant trend was found between PD and latitude when relative abundances and dominance were considered (**Figure 3C**, *q* = 1 and *q* = 2, respectively), which remained fairly constant along the latitudinal gradient. The decrease in phylogenetic entities and loss of the latitudinal pattern can be explained by the high contribution of rare species to diversity in species-rich tropical communities (Leitão et al., 2016; Lamanna et al., 2017; Cazzolla Gatti et al., 2022).

The presence and persistence of rare species is inversely correlated with latitude in tropical forests worldwide (Lamanna et al., 2017). In addition, the diversity of all species increases towards lower latitudes, but the diversity of rare species appears to increase more steeply than those of common or dominant species when approaching the Equator (Stevens & Willig, 2002; Lamanna et al., 2017). Thus, the loss of a significant latitudinal pattern for PD (*q* = 1 and *q* = 2) in the present study suggests important contributions of rare species to the maintenance of PD in western Amazonian *terra firme* forests (Leitão et al., 2016).

### Effects of environmental variables on TD, FD, and PD

The overall diversity tended to increase as the temperature seasonality decreased, annual precipitation increased, soil pH decreased, and soil bulk density decreased, although to a lesser extent for the latter (**Figure 4**). Temperature seasonality seemed to be the most important environmental driver for shaping diversity trends because it was included as a significant factor in all of the best-fit models for all three diversity attributes, where the only exception was PD with *q* = 2 (**Table 3**). Annual precipitation was also included in all of the models but it was a significant factor only for TD with *q* = 1 and *q* = 2, and PD with *q* = 0 (**Table 3**), thereby suggesting that seasonal temperature variation was more important for shaping diversity than total water discharge. Similarly, previous studies consistently found that temperature variables had greater power for explaining diversity patterns than precipitation variables (Giehl & Jarenkow, 2012; Moles et al., 2014; Chen et al., 2015). Our findings are also consistent with environmental filtering and the favourability hypothesis for all diversity attributes because diversity increases with lower temperature seasonality and higher precipitations (Fischer, 1960; Swenson et al., 2012b; Qian et al., 2013; Asefa et al., 2017; Wieczynski et al., 2019).

Among the edaphic factors, the soil pH was present in all of the best-fit models, except for TD with *q* = 2, and it was a significant fixed factor in the best-fit models for TD with *q* = 0 and *q* = 1, FD with *q* = 0, and PD with *q* = 0 and *q* = 1 (**Table 3**). Our results showed that the diversity was greater when the soil was more acidic, which suggests that these communities are adapted to high soil acidity conditions (pH 3.5–4.5), although soils with a pH under 5.3 are characterized by low availability of base metal cation and toxic aluminium concentrations (Sollins, 1998; Bañares de Dios et al., 2022). In addition, the soil bulk density only appeared in the best-fit models for TD with *q* = 0, and PD with *q* = 0 and *q* = 1 (**Table 3**). Diversity increased as the bulk density decreased and the soils had lower bulk density values of 0.47 to 1.11 g/cm^3^ than average ones for fully mineral soils, *i.e*., clay = 1.0 to 1.6 g/cm^3^ and sand = 1.2 to 1.8 g/cm^3^ (Athira et al., 2019), which suggests that the organic matter content increased as the bulk density decreased (Bauer, 1974; Athira et al., 2019; de la Cruz-Amo et al., 2020). Therefore, the soil organic matter content may have facilitated the adaptation of tropical plants to soils with low nutrient contents, high aluminium concentrations and low pH. Tropical soils are regarded as depleted of organic matter but large amounts of organic material are continuously supplied by decaying vegetation and its breakdown usually occurs very fast in the tropics, with rapid mineralization and availability for uptake (Herrera et al., 1978; Craswell & Lefroy, 2001; de la Cruz-Amo et al., 2020).

Overall, we found that TD increased as the temperature seasonality decreased, precipitation increased, and soil acidity increased (**Figure 4A**). Previous studies have demonstrated that the species richness of woody plants is negatively correlated with temperature seasonality, and positively correlated with the mean annual temperature and soil water content (Hofhansl et al., 2020). When we considered dominance (*q* = 2), only climatic factors were included in the best-fit model. This suggests that dominant species with wider tolerances and more widespread distributions were more dependent on the climate than the soils compared with rarer or less abundant species, which seemed more related to the local edaphic conditions (Pitman et al., 2013; Arellano et al., 2014).

According to our results, FD was affected by the temperature seasonality, annual precipitation and soil pH (**Figure 4B**), even when relative abundances and dominance were considered (*q* = 1 and *q* = 2, respectively). These results confirm that functional strategies are a consequence of environmental filtering because abiotic factors affect FD of both rare and common species in a similar manner. Therefore, although tropical climatic conditions are benign, they still strongly condition the successful functional strategies (Fortunel et al., 2014; Lamanna et al., 2014), thereby agreeing with the predictions of the favourability hypothesis in tropical environments. Recent studies obtained similar results for climatic and edaphic drivers (Swenson, 2012a; Molina-Venegas et al., 2018; Hofhansl et al., 2020).

We found that PD increased as the temperature seasonality decreased, precipitation increased, soil acidity increased, and bulk density decreased (**Figure 4C**). When traits are phylogenetically conserved, PD increases with decreasing environmental harshness, *i.e*., increasing temperature or precipitation (Qian et al., 2013; Satdichanh et al., 2015; Qian et al., 2017). Therefore, environmental filtering and the favourability hypothesis are consistent with our results for PD with *q* = 0 and *q* = 1. Recent studies also showed that climatic filtering and soil fertility shaped PD of woody angiosperms (Honorio Coronado et al., 2015; Kubota et al., 2018). The lack of a best-fit model other than the null model for PD with *q* = 2 could be explained by the fact that dominant clades probably appeared and expanded under conditioning by their dispersion abilities and historical factors which are not reflected in the actual environmental conditions (Pitman et al., 2001; Arellano et al., 2016).

## Conclusions

The latitudinal diversity gradient is explained by the environmental filtering principle and favourability hypothesis through climatic factors. Increases in temperature and precipitation seasonality are found in tropical regions towards higher latitudes (Fortunel et al., 2014), thereby leading to a noticeable latitudinal diversity gradient on a global scale but also within narrower latitudinal ranges such as the tropics. We found that temperature seasonality was still a relevant driver of all diversity attributes comprising TD, FD, and PD within a 10°C latitudinal gradient in the Neotropics. Therefore, our results support environmental filtering and the favourability hypothesis within hyperdiverse tropical regions.

In addition, the positive relationships found between the three diversity attributes indicate that if we take measures to protect and conserve TD, we also indirectly protect FD and PD, at least in western Amazonian *terra firme* forests. Furthermore, several studies found effects of TD, FD, and PD on productivity, and thus preserving forests with multiple functional strategies and a great evolutionary legacy will also protect their vital ecosystem functions (Balvanera et al., 2006; Gamfeldt et al., 2008; Cadotte, 2013; Coelho de Souza et al., 2019). Future studies should aim to understand diversity from this holistic perspective.

## Data availability statement

The raw data supporting the conclusions of this article will be made available by the authors, without undue reservation.

## Author contributions

CB, GB, MM and LC conceived the ideas. CB, JA and GB collected the data. NS and MTC provided bureaucratic, logistic and field support. CB and GB analyzed the data. CS led the writing. GB, JA, LM-G, LC, and MM gave continuous input to the manuscript writing and statistical analyses. All authors contributed to the article and approved the submitted version.

## Funding

The appropriate permits were obtained to collect material in the field: Área de Conservación Regional Maijuna-Kichwa No. 003-2019-GLR-GGR-ARA, Parque Nacional Río Abiseo No. 001-2016-SERNANP-PNRA-JEF, Zona de Amortiguamientó Parque Nacional Cordillera Azul No. 315-2017-SERFOR-DGGSPFFS, Zona de Amortiguamiento Reserva Comunal Yanesha No. 401-2018-MINAGRISERFOR-DGGSPFFS, Reserva Nacional Tambopata No. 35-2017-SERNANP-DGANP.

## Acknowledgments

We would like to express our most sincere gratitude to the native communities of Nueva Vida, Leoncio Prado, San Carlos, Yamino and Infierno for receiving us so kindly in their homes and forests, and for their crucial assistance in the field. We would also like to thank the Peruvian local authorities of Servicio Nacional de Áreas Naturales Protegidas por el Estado (SERNANP), Servicio Nacional Forestal (SERFOR), and Autoridad Regional Ambiental del Gobierno Regional de Loreto (ARA-GOREL) for assistance and support that made the expeditions possible in the sampled protected areas. We offer our most sincere gratitude to Victoria Cala, who performed the edaphic analyses, for her much appreciated contribution. We show our special appreciation to Luis Torres Montenegro for its valuable taxonomic and logistic work; Mara Paneghel for her crucial and most valuable help; Manuel Marca for his assistance in the field; Camino Monsalve for her contribution to the functional traits; and Iñigo Gómez, Maaike Pyck and Silvia Aguado for their much appreciated volunteer work.

## Conflict of interest

The authors declare that the research was conducted in the absence of any commercial or financial relationships that could be construed as a potential conflict of interest.

## Publisher’s note

All claims expressed in this article are solely those of the authors and do not necessarily represent those of their affiliated organizations, or those of the publisher, the editors and the reviewers. Any product that may be evaluated in this article, or claim that may be made by its manufacturer, is not guaranteed or endorsed by the publisher.
