# Supplementary figures and images for: Latitudinal patterns and environmental drivers of taxonomic, functional, and phylogenetic diversity of woody plants in western Amazonian terra firme forests
Source: Front Plant Sci. 2022 Oct 7;13:978299. doi: 10.3389/fpls.2022.978299 (PMC9585299; doi:10.3389/fpls.2022.978299)

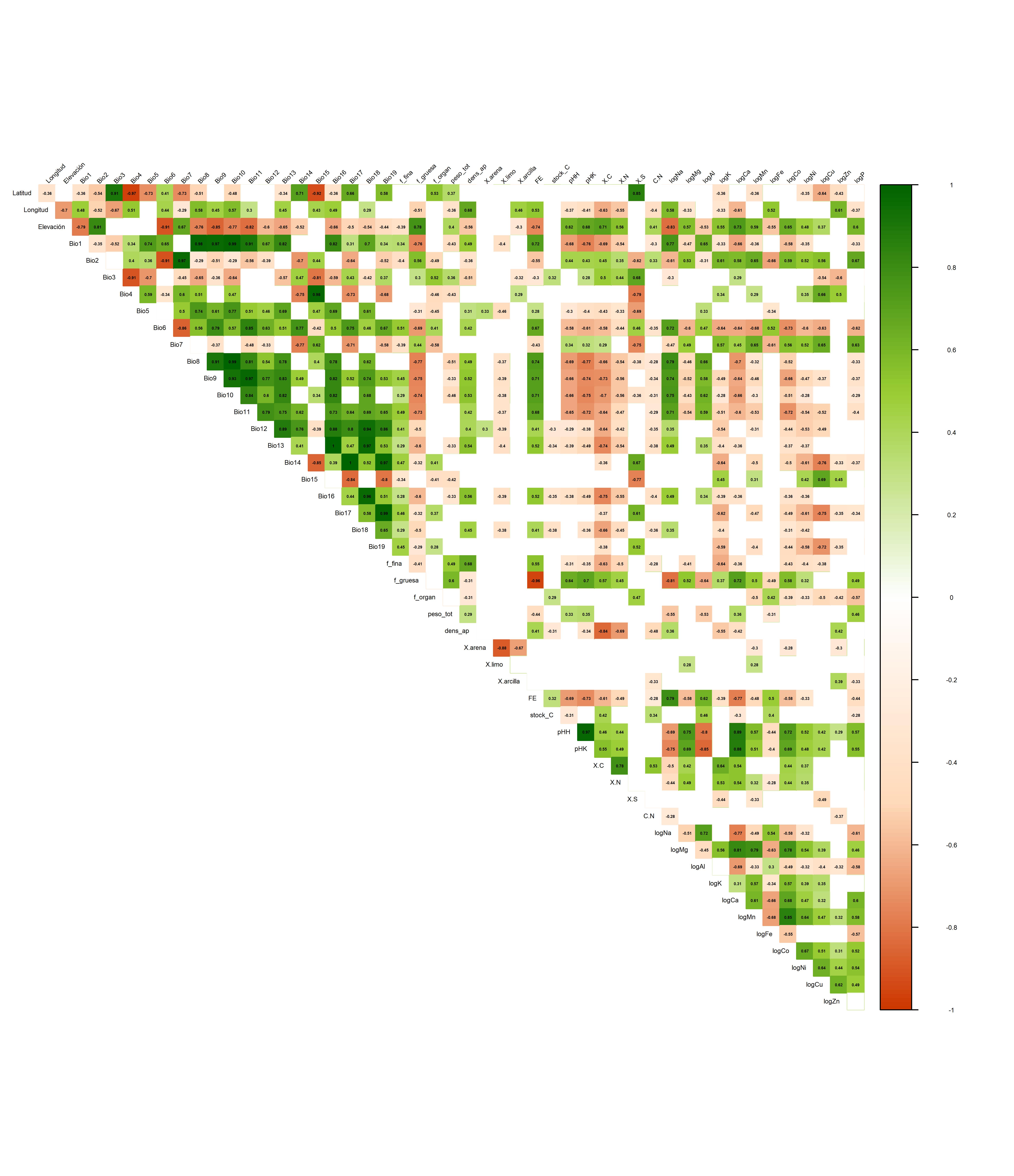

Supplement: Supplementary file 1 [file Image_1.jpeg]
