# Supplementary material for: Latitudinal patterns and environmental drivers of taxonomic, functional, and phylogenetic diversity of woody plants in western Amazonian terra firme forests
Source: Front Plant Sci. 2022 Oct 7;13:978299. doi: 10.3389/fpls.2022.978299 (PMC9585299; doi:10.3389/fpls.2022.978299)
Supplement: Supplementary file 2 [file Table_1.pdf]

SUPPLEMENTARY MATERIAL

TABLE S1. Coordinates, elevation and mean environmental data of the five studied regions in western Amazonia.

Study areas	Latitude	Longitude	Mean elevation (m.a.s.l.)	Temperature seasonality (°C)	Annual precipitation (mm/year)	Soil bulk density (g/cm^3)	Soil pH
Maijuna	-2.949611	-73.112723	121	4.16	2995	0.82	4.01
Rio Abiseo	-7.428176	-76.901853	881	5.95	1756	0.69	5.46
Cordillera Azul	-8.880584	-75.676112	343	6.06	3948	0.83	4.28
Yanesha	-10.224445	-75.316139	433	7.68	2146	0.61	3.78
Tambopata	-12.827000	- 69.275778	197	9.30	2701	1.00	4.09

TABLE S2. Acronym, definition and units of CHELSA bioclim variables.

Acronym	CHELSA bioclim variables	Units
Bio 1	Annual Mean Temperature	°C
Bio 2	Mean Diurnal Range	°C
Bio 3	Isothermality	°C
Bio 4	Temperature Seasonality	°C
Bio 5	Maximum Temperature of Warmest Month	°C
Bio 6	Minimum Temperature of Coldest Month	°C
Bio 7	Temperature Annual Range	°C
Bio 8	Mean Temperature of Wettest Quarter	°C
Bio 9	Mean Temperature of Driest Quarter	°C
Bio 10	Mean Temperature of Warmest Quarter	°C
Bio 11	Mean Temperature of Coldest Quarter	°C
Bio 12	Annual Precipitation	mm/year
Bio 13	Precipitation of Wettest Month	mm/month
Bio 14	Precipitation of Driest Month	mm/month
Bio 15	Precipitation Seasonality	mm/year
Bio 16	Precipitation of Wettest Quarter	mm/quarter
Bio 17	Precipitation of Driest Quarter	mm/quarter
Bio 18	Precipitation of Warmest Quarter	mm/quarter
Bio 19	Precipitation of Coldest Quarter	mm/quarter

TABLE S3. Description and units of the measured edaphic variables.

Edaphic variables	Description	Units
Bulk density	Total density of soil sample	g/cm^3
pHH	pH measure in water	pH
pHK	pH measure in KOH-	pH
% sand	Sand relative content in fine fraction	%
% silt	Silt relative content in fine fraction	%
% clay	Clay relative content in fine fraction	%
% C	Carbon relative content in fine fraction	%
% N	Nitrogen relative content in fine fraction	%
% S	Sulphur relative content in fine fraction	%
C/N	Ratio C/N in fine fraction	
Fine Earth	Ratio fine fraction/total weight	
C stock	Carbon stock	ton/ha
Na	Sodium content in fine fraction	mg/kg
Mg	Magnesium content in fine fraction	mg/kg
Al	Aluminium content in fine fraction	mg/kg
K	Potassium content in fine fraction	mg/kg
Ca	Calcium content in fine fraction	mg/kg
Mn	Manganese content in fine fraction	mg/kg
Fe	Iron content in fine fraction	mg/kg
Co	Cobalt content in fine fraction	mg/kg
Ni	Nickel content in fine fraction	mg/kg
Cu	Copper content in fine fraction	mg/kg
Zn	Zinc content in fine fraction	mg/kg
P	Phosphorus content in fine fraction	mg/kg

FIGURE S1. Pearson's correlation between environmental variables, including latitude.

TABLE S4. Pearson's correlation values between the selected environmental factors analysed in 50 0.1 ha plots in western Amazonia. Significance levels represented by: (*) when p<0.05, (**) when p<0.01 and (***) when p<0.001.

Environmental factors	Latitude	Temperature seasonality	Annual precipitation	Soil bulk density
**Temperature seasonality**	-0.97***			
**Annual precipitation**	0.09	-0.21		
**Soil bulk density**	-0.20	0.25	0.40**	
**Soil pH**	0.11	-0.18	-0.29*	-0.26

TABLE S6. Sampled individuals, number of taxa, mean abundance and species richness per plot, number of taxa with functional, taxonomical and phylogenetic information for the five studied regions.

Study areas	Nº individuals	Nº taxa	Mean abundance per plot	Mean species richness per plot	Nº taxa with all functional traits	Nº taxa with taxonomic and phylogenetic data
Maijuna	2963	621	259	140	450 (72%)	542 (87%)
Rio Abiseo	2770	355	277	100	269 (76%)	292 (82%)
Cordillera Azul	3131	599	265	127	466 (78%)	511 (85%)
Yanesha	3262	535	300	122	442 (83%)	467 (87%)
Tambopata	2555	343	237	86	274 (80%)	302 (88%)
