# Supplementary material for: Latitudinal patterns and environmental drivers of taxonomic, functional, and phylogenetic diversity of woody plants in western Amazonian terra firme forests
Source: Front Plant Sci. 2022 Oct 7;13:978299. doi: 10.3389/fpls.2022.978299 (PMC9585299; doi:10.3389/fpls.2022.978299)
Supplement: Supplementary file 3 [file Table_2.pdf]

Table S5. List of species found in the studied areas in western Amazonia with records for all functional traits. Mean values of LT (Leaf Thickness), SLA (Specific Leaf Area), and WD (Wood Density) shown for each species.

Taxon	LT (mm)	SLA (mm^2/mg)	WD (g/cm^3)
Abarema auriculata	0,08	21,15	0,34
Abarema laeta	0,10	24,05	0,61
Abuta grandifolia	0,13	11,84	0,53
Abuta rufescens	0,10	10,74	0,65
Acalypha cuneata	0,09	23,02	0,60
Adenocalymma bracteolatum	0,11	11,03	0,56
Adenocalymma impressum	0,18	17,13	0,42
Adenocalymma inundatum	0,13	15,87	0,55
Aegiphila smithii	0,05	40,26	0,26
Aegiphila sufflava	0,13	20,30	0,29
Aegiphila ulei	0,08	36,83	0,44
Agonandra silvatica	0,17	23,72	0,57
Agouticarpa curviflora	0,15	10,41	0,81
Aiouea grandifolia	0,14	14,65	0,61
Alchornea glandulosa	0,07	21,36	0,36
Alchornea triplinervia	0,08	21,34	0,24
Alchorneopsis floribunda	0,18	13,26	0,40
Alibertia claviflora	0,11	12,78	0,70
Allantoma decandra	0,11	15,00	0,57
Allantoma pluriflora	0,06	16,40	0,51
Allophylus divaricatus	0,11	24,19	0,38
Allophylus floribundus	0,11	22,11	0,32
Allophylus paniculatus	0,06	22,47	0,60
Allophylus pilosus	0,07	33,18	0,36
Alseis lugonis	0,08	23,51	0,40
Amaioua glomerulata	0,14	13,73	0,67
Amaioua guianensis	0,12	14,55	0,71
Ambelania occidentalis	0,20	10,19	0,67
Ampelocera edentula	0,16	12,85	0,56
Amphilophium dasytrichum	0,10	17,66	0,43
Anacardium giganteum	0,13	16,77	0,41
Anaxagorea brachycarpa	0,11	14,37	0,50
Anaxagorea dolichocarpa	0,15	12,38	0,48
Anaxagorea phaeocarpa	0,10	16,70	0,56
Andira inermis	0,15	17,65	0,36
Andira multistipula	0,09	22,87	0,50
Andira vermifuga	0,08	21,19	0,55
Anemopaegma oligoneuron	0,10	24,81	0,46
Aniba canelilla	0,14	13,73	0,61
Aniba firmula	0,10	17,57	0,49
Aniba guianensis	0,18	10,78	0,62
Aniba hostmanniana	0,15	13,54	0,57
Aniba megaphylla	0,15	9,92	0,61
Aniba panurensis	0,19	11,65	0,63
Aniba perutilis	0,16	9,61	0,53
Aniba puchury-minor	0,12	14,19	0,58
Aniba riparia	0,19	13,09	0,47
Aniba rosaeodora	0,23	10,89	0,44
Aniba taubertiana	0,16	11,15	0,68
Aniba taubertiana	0,19	10,63	0,58
Aniba williamsii	0,27	7,10	0,54
Anisophyllea guianensis	0,15	13,53	0,79
Annona cherimolioides	0,10	23,52	0,58
Annona hypoglauca	0,09	15,67	0,37
Annona montana	0,20	18,44	0,48
Annona papilionella	0,13	19,08	0,19
Anomospermum bolivianum	0,21	14,08	0,38
Anomospermum grandifolium	0,20	9,87	0,37
Anomospermum solimoesianum	0,08	12,66	0,36
Anthodiscus klugii	0,13	12,88	0,59
Apeiba glabra	0,10	24,63	0,35
Apuleia leiocarpa	0,11	24,83	0,48
Ardisia pellucida	0,11	14,41	0,55
Aristolochia ruiziana	0,03	37,29	0,40
Aspidosperma excelsum	0,19	14,61	0,57
Aspidosperma parvifolium	0,12	20,83	0,59
Aspidosperma spruceanum	0,25	8,02	0,66
Astronium lecointei	0,06	11,61	0,42
Banara guianensis	0,07	35,53	0,46
Banisteriopsis elegans	0,18	9,22	0,35
Batocarpus costaricensis	0,06	17,03	0,48

Batocarpus orinocensis	0,12	19,70	0,53
Bauhinia brachycalyx	0,08	17,21	0,56
Bauhinia glabra	0,09	33,92	0,29
Bauhinia longicuspis	0,08	18,61	0,49
Beilschmiedia pendula	0,12	16,11	0,44
Bellucia pentamera	0,17	10,04	0,64
Bellucia subandina	0,13	21,45	0,55
Bertholletia excelsa	0,09	14,51	0,50
Bignonia nocturna	0,15	19,39	0,49
Bignonia sciuripabulum	0,15	23,22	0,30
Borismene japurensis	0,22	7,54	0,47
Brosimum acutifolium	0,09	24,13	0,42
Brosimum guianense	0,10	19,79	0,56
Brosimum lactescens	0,14	12,46	0,60
Brosimum rubescens	0,14	14,35	0,64
Brosimum utile	0,15	12,47	0,44
Brownea cauliflora	0,08	12,90	0,59
Brownea grandiceps	0,12	8,30	0,62
Brownea ucayalina	0,09	14,48	0,62
Buchenavia macrophylla	0,13	16,17	0,65
Buchenavia oxycarpa	0,09	24,55	0,57
Bunchosia argentea	0,15	21,10	0,46
Bunchosia armeniaca	0,14	19,36	0,55
Bunchosia cauliflora	0,08	21,32	0,36
Bunchosia hookeriana	0,14	22,47	0,55
Byrsonima arthropoda	0,14	14,79	0,43
Byrsonima japurensis	0,09	15,55	0,54
Byrsonima poeppigiana	0,18	12,79	0,50
Byrsonima putumayensis	0,12	17,32	0,33
Byttneria asterotricha	0,10	23,82	0,37
Cabralea canjerana	0,09	12,57	0,19
Calatola costaricensis	0,17	22,57	0,43
Calliandra glyphoxylon	0,07	17,11	0,62
Callichlamys latifolia	0,08	11,48	0,58
Calophyllum brasiliense	0,14	15,88	0,65
Calycophyllum megistocaulum	0,15	11,15	0,45
Calyptranthes macrophylla	0,23	13,37	0,75
Calyptranthes speciosa	0,17	9,88	0,61
Capirona decorticans	0,24	14,43	0,42
Capparidastrum sola	0,13	15,53	0,67
Caraipa densifolia	0,15	11,77	0,54
Carapichea adinantha	0,14	14,67	0,61
Carapichea affinis	0,23	15,35	0,42
Carpotroche longifolia	0,11	14,75	0,41
Caryocar glabrum	0,06	15,25	0,28
Caryodaphnopsis fosteri	0,08	15,73	0,55
Caryodaphnopsis inaequalis	0,11	17,22	0,55
Caryodendron grandifolium	0,13	17,68	0,59
Casearia aculeata	0,10	33,20	1,09
Casearia arborea	0,12	15,86	0,55
Casearia javitensis	0,14	20,05	0,57
Casearia maynacarpa	0,08	12,09	0,66
Casearia pitumba	0,08	30,71	0,48
Casearia ulmifolia	0,11	31,79	0,39
Cassia swartzioides	0,09	11,13	0,48
Castilla ulei	0,07	30,99	0,38
Cathedra acuminata	0,18	24,24	0,61
Cecropia concolor	0,13	6,36	0,40
Cecropia engleriana	0,15	7,37	0,40
Cecropia membranacea	0,07	23,26	0,22
Cecropia sciadophylla	0,15	6,64	1,13
Cedrela odorata	0,12	13,05	0,43
Cedrelinga cateniformis	0,18	13,48	0,60
Ceiba pentandra	0,10	24,84	0,33
Ceiba samauma	0,06	29,15	0,20
Celtis schippii	0,11	19,04	0,49
Cestrum schlechtendahlii	0,15	17,57	0,52
Cheiloclinium anomalum	0,22	9,66	0,67
Cheiloclinium articulatum	0,11	19,93	0,60
Cheiloclinium cognatum	0,14	11,86	0,73
Cheiloclinium hippocrateoides	0,12	14,52	0,58
Chimarrhis glabriflora	0,29	9,90	0,48
Chimarrhis hookeri	0,09	21,20	0,37
Chionanthus chrysopetalus	0,10	20,45	0,64
Chrysochlamys membranacea	0,22	17,83	0,49

Chrysochlamys ulei	0,26	13,68	0,47
Chrysophyllum amazonicum	0,13	13,33	0,39
Chrysophyllum argenteum	0,11	16,74	0,66
Chrysophyllum bombycinum	0,15	8,71	0,44
Chrysophyllum colombianum	0,08	18,81	0,66
Chrysophyllum manaosense	0,11	11,64	0,45
Chrysophyllum prieurii	0,26	6,17	0,65
Chrysophyllum sanguinolentum	0,14	9,79	0,52
Cissus pseudofuliginea	0,15	16,12	0,34
Clarisia biflora	0,11	16,96	0,41
Clarisia racemosa	0,10	18,87	0,53
Clavija poeppigii	0,17	10,43	0,37
Clitoria pozuzoensis	0,08	23,75	0,41
Clusia amazonica	0,30	17,15	0,43
Clusia grandiflora	0,55	3,69	0,48
Clusia hammeliana	0,29	13,46	0,62
Clusia microstemon	0,23	5,06	0,52
Clusia octandra	0,37	7,23	0,61
Coccoloba densifrons	0,18	13,45	0,40
Coccoloba marginata	0,16	15,90	0,56
Coccoloba padiformis	0,16	11,89	0,50
Coccoloba paraensis	0,11	12,33	0,59
Colubrina spinosa	0,16	17,12	0,47
Combretum laxum	0,12	12,40	0,72
Compsoneura capitellata	0,11	15,11	0,49
Compsoneura sprucei	0,12	18,66	0,39
Conceveiba guianensis	0,10	13,39	0,51
Conceveiba martiana	0,12	31,77	0,41
Conceveiba rhytidocarpa	0,15	11,07	0,61
Conceveiba terminalis	0,17	10,11	0,49
Connarus fasciculatus	0,12	12,81	0,52
Connarus wurdackii	0,18	11,51	0,64
Copaifera paupera	0,16	7,78	0,47
Cordia lomatoloba	0,12	21,32	0,37
Cordia nodosa	0,10	18,28	0,27
Cordia panamensis	0,14	30,27	0,28
Cordia scabrifolia	0,19	11,60	0,35
Cordia sprucei	0,16	22,06	0,33
Cordia ucayaliensis	0,17	16,69	0,31
Cordia ulei	0,26	6,73	0,38
Couepia chrysocalyx	0,11	10,84	0,78
Couepia krukovii	0,15	13,39	0,60
Couepia macrophylla	0,16	10,49	0,64
Couepia paraensis	0,13	10,04	0,62
Couepia parillo	0,14	9,70	0,80
Couepia williamsii	0,14	14,36	0,71
Couma macrocarpa	0,14	13,43	0,30
Couratari guianensis	0,10	17,27	0,45
Couratari macrosperma	0,15	13,80	0,46
Coussapoa tessmannii	0,37	5,96	0,52
Coussapoa trinervia	0,13	8,89	0,60
Coussapoa villosa	0,32	6,28	0,28
Coussarea macrophylla	0,17	16,27	0,54
Coussarea tortilis	0,20	15,91	0,46
Cremastosperma cauliflorum	0,15	13,38	0,47
Cremastosperma cenepense	0,14	11,97	0,48
Cremastosperma leiophyllum	0,09	18,83	0,52
Cremastosperma oblongum	0,13	12,80	0,43
Crepidospermum goudotianum	0,12	20,95	0,43
Crepidospermum prancei	0,07	18,87	0,48
Crepidospermum rhoifolium	0,12	11,57	0,54
Croton schiedeanus	0,13	17,77	0,57
Croton tessmannii	0,15	14,33	0,54
Cuervea kappleriana	0,16	12,61	0,60
Cupania cinerea	0,11	10,72	0,44
Curarea tecunarum	0,12	8,21	0,49
Curarea toxicofera	0,22	9,90	0,45
Cybianthus comperuvianus	0,20	12,80	0,65
Cybianthus gigantophyllus	0,14	11,82	0,60
Cymbopetalum longipes	0,14	24,53	0,46
Dacryodes amplectans	0,11	11,45	0,41
Dacryodes chimantensis	0,29	7,15	0,56
Dacryodes hopkinsii	0,14	8,91	0,52
Dacryodes nitens	0,18	7,64	0,50
Dacryodes peruviana	0,14	11,07	0,53

Dalbergia frutescens	0,15	2,69	0,33
Dalbergia monetaria	0,10	14,82	0,55
Dalbergia monetaria	0,18	10,95	0,64
Damburneya purpurea	0,12	24,95	0,41
Davilla kunthii	0,28	8,88	0,58
Deguelia scandens	0,09	15,49	0,55
Dendrobangia boliviana	0,17	19,20	0,54
Dendrobangia multinervia	0,10	25,83	0,51
Dendropanax arboreus	0,19	17,00	0,48
Dendropanax macropodus	0,23	12,48	0,32
Dialium guianense	0,12	23,21	0,61
Diclinanona calycina	0,06	21,98	0,42
Diclinanona tessmannii	0,16	17,11	0,54
Dicranostyles ampla	0,15	13,36	0,60
Dicranostyles globostigma	0,12	13,17	0,54
Dicranostyles holostyla	0,09	17,50	0,69
Dicranostyles longifolia	0,13	14,58	0,53
Dicranostyles scandens	0,14	17,74	0,52
Dilkea cuneata	0,17	11,74	0,59
Dilkea retusa	0,12	16,39	0,63
Diospyros artanthifolia	0,13	12,53	0,48
Diospyros capreifolia	0,11	15,49	0,47
Diospyros subrotata	0,17	12,68	0,67
Diospyros tessmannii	0,15	11,21	0,63
Diploon cuspidatum	0,13	13,47	0,73
Diplotropis purpurea	0,08	14,91	0,57
Dipteryx odorata	0,13	14,12	0,68
Discophora guianensis	0,13	15,42	0,41
Distictella racemosa var. translucida	0,10	16,48	0,35
Doliocarpus amazonicus	0,18	11,30	0,46
Doliocarpus dentatus	0,11	18,86	0,31
Drypetes amazonica	0,12	13,42	0,70
Drypetes gentryana	0,14	13,27	0,68
Drypetes gentryi	0,18	8,25	0,71
Duguetia flagellaris	0,14	18,94	0,62
Duguetia hadrantha	0,14	13,58	0,61
Duguetia macrophylla	0,23	11,19	0,51
Duguetia odorata	0,13	11,95	0,56
Duguetia quitarensis	0,13	15,78	0,60
Duguetia spixiana	0,13	14,41	0,59
Duguetia trunciflora	0,16	14,16	0,56
Dulacia candida	0,17	18,76	0,54
Dulacia guianensis	0,18	24,31	0,29
Duroia hirsuta	0,10	18,14	0,47
Dussia foxii	0,13	7,35	0,51
Ecclinusa guianensis	0,12	8,77	0,54
Ecclinusa lanceolata	0,10	11,88	0,55
Elachyptera floribunda	0,12	13,38	0,55
Endlicheria arachnocome	0,14	14,22	0,56
Endlicheria bracteata	0,06	20,31	0,47
Endlicheria directonervia	0,17	14,05	0,33
Endlicheria formosa	0,22	7,75	0,45
Endlicheria griseosericea	0,10	13,97	0,35
Endlicheria klugii	0,18	10,30	0,48
Endlicheria krukovii	0,14	14,99	0,41
Endlicheria macrophylla	0,13	11,18	0,53
Endlicheria metallica	0,19	10,73	0,44
Endlicheria pyriformis	0,18	10,84	0,29
Endlicheria robusta	0,17	12,44	0,39
Endlicheria ruforamula	0,17	12,16	0,32
Endlicheria sprucei	0,14	13,93	0,70
Endlicheria verticillata	0,12	14,65	0,57
Enterolobium barnebianum	0,07	19,69	0,35
Erythroxylum fimbriatum	0,16	17,60	0,61
Erythroxylum macrophyllum	0,13	15,95	0,63
Erythroxylum mucronatum	0,14	13,23	0,72
Eschweilera albiflora	0,15	8,57	0,65
Eschweilera andina	0,14	10,90	0,57
Eschweilera apiculata	0,10	16,26	0,65
Eschweilera bracteosa	0,16	14,48	0,66
Eschweilera chartaceifolia	0,12	13,39	0,56
Eschweilera coriacea	0,15	12,33	0,64
Eschweilera decolorans	0,21	7,41	0,67
Eschweilera gigantea	0,19	9,36	0,67
Eschweilera grandiflora	0,22	9,13	0,66

Eschweilera itayensis	0,13	12,18	0,60
Eschweilera laevicarpa	0,18	11,15	0,49
Eschweilera ovalifolia	0,11	15,55	0,71
Eschweilera parvifolia	0,16	13,61	0,62
Eschweilera tessmannii	0,17	10,33	0,75
Eugenia feijoi	0,16	12,53	0,70
Eugenia florida	0,08	18,98	0,58
Eugenia gomesiana	0,19	9,85	0,64
Eugenia scalariformis	0,14	10,93	0,43
Eugenia schunkei	0,29	5,82	0,74
Eugenia uniflora	0,12	17,63	0,56
Faramea anisocalyx	0,09	24,74	0,57
Faramea capillipes	0,12	35,82	0,56
Faramea condorica	0,08	23,56	0,51
Faramea glandulosa	0,16	20,43	0,50
Faramea quinqueflora	0,09	31,26	0,61
Faramea tamberlikiana	0,18	13,54	0,55
Faramea torquata	0,16	12,20	0,60
Ferdinandusa guainiae	0,16	13,69	0,51
Ferdinandusa loretensis	0,19	11,24	0,48
Fevillea cordifolia	0,15	18,09	0,26
Ficus americana subsp. guianensis	0,18	8,47	0,47
Ficus crocata	0,18	11,35	0,41
Ficus gomelleira	0,09	23,84	0,72
Ficus insipida	0,18	10,22	0,40
Ficus maxima	0,11	21,18	0,37
Ficus maximoides	0,11	19,03	0,41
Ficus paraensis	0,11	28,05	0,38
Ficus pertusa	0,17	15,31	0,53
Ficus pulchella	0,24	11,16	0,48
Ficus schippii	0,20	13,40	0,41
Ficus trigona	0,15	12,83	0,34
Forsteronia affinis	0,08	24,13	0,47
Forsteronia graciloides	0,06	23,57	0,41
Fridericia cinnamomea	0,08	15,27	0,68
Fridericia japurensis	0,03	45,37	0,47
Fridericia pearcei	0,10	21,32	0,31
Fridericia trailii	0,18	9,00	0,63
Froesia diffusa	0,08	8,48	0,18
Froesiodendron longicuspe	0,13	23,56	0,26
Fusaea longifolia	0,14	11,74	0,57
Fusaea peruviana	0,12	11,30	0,51
Fusispermum laxiflorum	0,16	15,49	0,42
Garcinia intermedia	0,24	6,92	0,70
Garcinia macrophylla	0,23	8,60	0,71
Garcinia madruno	0,22	12,69	0,66
Gaulettia canomensis	0,16	14,64	0,77
Gloeospermum equatoriense	0,11	26,75	0,56
Glycydendron amazonicum	0,10	20,67	0,49
Gnetum nodiflorum	0,27	7,52	0,41
Gouania aptera	0,07	36,20	0,28
Goupia glabra	0,11	19,18	0,59
Guapira noxia	0,17	14,20	0,43
Guarea cristata	0,17	12,48	0,53
Guarea gomma	0,16	11,39	0,43
Guarea grandifolia	0,16	7,15	0,40
Guarea kunthiana	0,24	9,40	0,47
Guarea macrophylla	0,14	17,98	0,56
Guarea pterorhachis	0,15	5,55	0,53
Guarea pubescens	0,12	19,77	0,65
Guarea scabra	0,12	12,78	0,65
Guarea silvatica	0,15	17,80	0,47
Guarea trunciflora	0,13	9,82	0,58
Guatteria decurrens	0,15	19,91	0,45
Guatteria guianensis	0,21	8,80	0,49
Guatteria hirsuta	0,13	18,67	0,39
Guatteria megalophylla	0,16	10,42	0,43
Guatteria modesta	0,13	18,70	0,44
Guatteria punctata	0,15	11,47	0,32
Guatteria ramiflora	0,14	10,47	0,32
Guatteria scytophylla	0,13	16,05	0,30
Guatteria ucayalina	0,10	14,80	0,36
Gurania acuminata	0,24	13,12	0,45
Gustavia augusta	0,12	11,34	0,55
Gustavia hexapetala	0,16	11,19	0,62

Handroanthus serratifolius	0,04	36,74	0,57
Hasseltia floribunda	0,13	20,35	0,42
Hebepetalum humiriifolia	0,12	20,93	0,64
Heisteria acuminata	0,12	16,03	0,57
Heisteria insculpta	0,15	8,27	0,73
Heisteria scandens	0,08	23,19	0,57
Heisteria spruceana	0,13	13,01	0,72
Helicostylis scabra	0,07	19,17	0,44
Helicostylis tomentosa	0,12	17,07	0,54
Helicostylis turbinata	0,13	20,93	0,43
Herrania nitida	0,04	19,07	0,28
Heteropterys prancei	0,20	14,54	0,52
Hevea guianensis	0,08	30,12	0,28
Hieronyma alchorneoides	0,08	19,53	0,29
Hieronyma oblonga	0,20	23,21	0,47
Himatanthus phagedaenicus	0,08	32,63	0,53
Himatanthus tarapotensis	0,11	22,13	0,43
Hippocratea volubilis	0,12	15,16	0,52
Hippotis triflora	0,08	34,47	0,46
Hiraea fagifolia	0,14	14,56	0,30
Hiraea grandifolia	0,16	19,64	0,88
Hirtella bicornis	0,11	20,18	0,67
Hirtella bullata	0,14	13,35	0,77
Hirtella excelsa	0,11	15,55	0,67
Hirtella hispidula	0,10	16,26	0,66
Hirtella pilosissima	0,06	15,67	0,57
Hirtella racemosa	0,13	13,61	0,73
Hirtella rodriguesii	0,09	20,75	0,69
Hirtella triandra	0,14	13,32	0,76
Huberodendron swietenioides	0,12	15,88	0,43
Hymenaea courbaril	0,19	12,35	0,70
Hymenaea oblongifolia	0,11	16,56	0,54
Hymenopus arachnoideus	0,13	10,81	0,77
Hymenopus heteromorphus	0,17	11,81	0,55
Hymenopus latifolius	0,10	12,32	0,67
Hymenopus prismatocarpus	0,16	8,82	0,71
Hymenopus reticulatus	0,11	13,33	0,70
Inga acicularis	0,19	12,77	0,70
Inga acreana	0,10	16,16	0,51
Inga acrocephala	0,15	13,28	0,45
Inga alba	0,07	17,04	0,53
Inga aliena	0,08	17,38	0,71
Inga auristellae	0,14	15,67	0,62
Inga bourgonii	0,11	12,74	0,52
Inga capitata	0,15	10,92	0,58
Inga cayennensis	0,09	14,43	0,52
Inga chartacea	0,15	11,72	0,55
Inga cordatoalata	0,11	15,16	0,60
Inga coruscans	0,12	17,65	0,53
Inga fastuosa	0,17	9,54	0,46
Inga gracilifolia	0,08	19,50	0,60
Inga heterophylla	0,08	14,81	0,53
Inga ilta	0,19	8,98	0,48
Inga ingoides	0,11	18,52	0,28
Inga japurensis	0,12	10,46	0,53
Inga leiocalycina	0,12	15,62	0,45
Inga macrophylla	0,14	13,55	0,26
Inga marginata	0,15	8,68	0,59
Inga megaphylla	0,15	10,43	0,52
Inga nobilis	0,10	13,50	0,64
Inga nobilis subsp. quaternata	0,14	9,02	0,58
Inga ruiziana	0,13	10,96	0,46
Inga sapindoides	0,11	18,88	0,64
Inga setosa	0,11	20,85	0,66
Inga stenoptera	0,11	19,51	0,52
Inga stipularis	0,13	14,91	0,53
Inga tenuistipula	0,10	15,56	0,61
Inga thibaudiana	0,12	10,49	0,46
Inga umbellifera	0,14	14,30	0,44
Inga venusta	0,15	7,85	0,36
Inga yasuniana	0,09	21,05	0,56
Iryanthera juruensis	0,14	14,12	0,50
Iryanthera laevis	0,14	14,29	0,67
Iryanthera lancifolia	0,22	9,30	0,46
Iryanthera macrophylla	0,25	8,12	0,51

Iryanthera paradoxa	0,22	8,46	0,38
Iryanthera paraensis	0,16	12,28	0,53
Iryanthera ulei	0,19	9,31	0,48
Ixora acuminatissima	0,19	12,10	0,85
Ixora peruviana	0,17	12,03	0,74
Ixora yavitensis	0,13	12,88	0,71
Jacaranda copaia	0,08	20,96	0,28
Jacaratia digitata	0,12	34,87	0,11
Joosia dichotoma	0,12	19,99	0,47
Klarobelia napoensis	0,06	15,25	0,64
Kutchubaea sericantha	0,16	8,83	0,54
Lacistema aggregatum	0,16	16,78	0,59
Lacistema nena	0,16	17,98	0,48
Lacistema pubescens	0,21	9,55	0,64
Lacmellea aculeata	0,13	20,55	0,51
Lacmellea ramosissima	0,12	20,26	0,45
Lacunaria jenmanii	0,07	13,99	0,49
Lacunaria macrostachya	0,09	25,12	0,65
Ladenbergia graciliflora	0,26	10,01	0,49
Ladenbergia oblongifolia	0,27	12,91	0,49
Laetia procera	0,11	18,98	0,59
Lecointea peruviana	0,15	10,71	0,79
Leonia crassa	0,20	9,39	0,50
Leonia cymosa	0,07	27,71	0,59
Leonia glycycarpa	0,17	12,68	0,49
Leptobalanus apetalus	0,09	13,33	0,63
Leptobalanus longistylus	0,17	12,91	0,60
Leptobalanus octandrus	0,11	15,77	0,60
Leretia cordata	0,10	19,35	0,51
Licania canescens	0,21	10,70	0,85
Licania ferreirae	0,16	10,43	0,72
Licania harlingii	0,09	17,38	0,66
Licania hypoleuca	0,09	19,13	0,77
Licania micrantha	0,21	8,15	0,68
Licania micrantha	0,12	10,62	0,67
Licania octandra	0,11	14,23	0,69
Licania pallida	0,13	14,97	0,70
Licania triandra	0,13	19,93	0,64
Licaria armeniaca	0,06	17,67	0,62
Licaria aurea	0,13	14,78	0,62
Licaria brasiliensis	0,09	16,03	0,65
Licaria cannella	0,09	14,34	0,49
Licaria macrophylla	0,18	22,14	0,56
Licaria pucheri	0,14	11,16	0,56
Lindackeria paludosa	0,10	16,22	0,49
Lonchocarpus densiflorus	0,09	13,14	0,41
Lonchocarpus seorsus	0,12	16,03	0,35
Lunania parviflora	0,10	27,90	0,46
Mabea angularis	0,14	12,35	0,62
Mabea excelsa	0,14	11,26	0,36
Mabea piriri	0,07	17,75	0,45
Mabea speciosa	0,12	13,39	0,63
Machaerium complanatum	0,12	26,90	0,51
Machaerium cuspidatum	0,10	24,37	0,52
Machaerium floribundum	0,07	19,95	0,42
Machaerium inundatum	0,07	22,92	0,59
Machaerium mutisii	0,17	9,49	0,68
Machaerium quinata	0,09	19,79	0,49
Macoubea sprucei	0,09	24,60	0,38
Macrolobium angustifolium	0,11	15,08	0,52
Macrolobium arenarium	0,10	15,08	0,56
Macrolobium gracile	0,07	16,11	0,56
Macrolobium klugii	0,27	8,67	0,57
Macrolobium limbatum	0,13	11,96	0,47
Magnolia amazonica	0,20	7,29	0,43
Manilkara inundata	0,13	15,96	0,70
Maprounea guianensis	0,08	46,21	0,51
Maquira calophylla	0,24	10,37	0,35
Maquira guianensis	0,12	20,58	0,55
Marcgravia atropunctata	0,17	8,64	0,58
Margaritaria nobilis	0,04	45,44	0,47
Marila pluricostata	0,11	12,99	0,55
Marila tomentosa	0,08	16,95	0,51
Mascagnia divaricata	0,15	17,97	0,67
Mascagnia ovatifolia	0,12	15,70	0,57

Matayba adenanthera	0,09	9,66	0,63
Matayba macrostylis	0,11	11,68	0,49
Matayba purgans	0,12	13,28	0,54
Matayba scrobiculata	0,12	18,34	0,67
Matisia bracteolosa	0,13	18,48	0,54
Matisia cordata	0,12	17,13	0,21
Matisia lecythicarpa	0,10	17,47	0,59
Matisia malacocalyx	0,12	18,14	0,46
Matisia ochrocalyx	0,11	20,77	0,43
Maytenus macrocarpa	0,11	11,56	0,67
Meliosma boliviensis	0,16	16,13	0,30
Meliosma caballeroensis	0,14	20,00	0,28
Meliosma frondosa	0,17	8,97	0,63
Meliosma herbertii	0,22	13,13	0,39
Mezilaurus opaca	0,18	8,14	0,78
Mezilaurus palcazuensis	0,16	9,30	0,77
Miconia affinis	0,13	15,09	0,60
Miconia amazonica	0,10	20,11	0,42
Miconia appendiculata	0,11	16,58	0,43
Miconia astroplocama	0,15	12,94	0,76
Miconia calvescens	0,12	19,49	0,34
Miconia cazaletii	0,10	26,08	0,49
Miconia chrysophylla	0,16	16,24	0,68
Miconia dolichorrhyncha	0,08	17,65	0,47
Miconia elaeagnoides	0,11	12,71	0,56
Miconia emendata	0,09	31,69	0,62
Miconia lepidota	0,17	8,07	0,67
Miconia membranacea	0,12	2,57	0,53
Miconia minutiflora	0,20	9,63	0,60
Miconia poeppigii	0,16	7,51	0,55
Miconia prasina	0,10	16,95	0,58
Miconia punctata	0,15	11,48	0,51
Miconia pyrifolia	0,09	13,53	0,53
Miconia semisterilis	0,16	14,29	0,55
Miconia serialis	0,10	15,33	0,68
Miconia stelligera	0,08	14,50	0,50
Miconia tomentosa	0,15	13,56	0,50
Miconia traillii	0,07	19,91	0,58
Miconia trinervia	0,19	13,92	0,43
Micropholis cylindrocarpa	0,11	15,07	0,60
Micropholis egensis	0,18	12,16	0,56
Micropholis guyanensis	0,14	12,27	0,65
Micropholis guyanensis subsp. duckeana	0,13	7,22	0,63
Micropholis madeirensis	0,15	11,20	0,66
Micropholis mensalis	0,14	12,24	0,58
Micropholis trunciflora	0,21	9,31	0,66
Micropholis venulosa	0,13	17,89	0,55
Micropholis williamii	0,10	13,18	0,69
Mimosa guilandinae	0,08	20,28	0,41
Minquartia guianensis	0,12	12,75	0,59
Mollia gracilis	0,12	16,94	0,48
Mollia lepidota	0,11	12,16	0,45
Mollinedia killipii	0,25	15,48	0,52
Mollinedia lanceolata	0,14	20,25	0,41
Mollinedia ovata	0,17	23,63	0,48
Moquilea brittoniana	0,13	14,70	0,72
Moquilea macrocarpa	0,14	11,20	0,54
Moquilea tomentosa	0,11	16,58	0,71
Mouriri acutiflora	0,21	10,55	0,80
Mouriri apiranga	0,16	10,54	0,63
Mouriri cauliflora	0,16	9,32	0,79
Mouriri grandiflora	0,23	9,95	0,76
Mouriri myrtifolia	0,11	14,96	0,84
Mouriri nigra	0,16	8,91	0,80
Myrcia aliena	0,14	15,14	0,63
Myrcia ambivalens	0,11	13,13	0,66
Myrcia deflexa	0,10	16,31	0,72
Myrcia densiflora	0,18	11,01	0,69
Myrcia dichasialis	0,17	13,21	0,48
Myrcia fallax	0,10	20,85	0,72
Myrcia huallagae	0,13	20,33	0,79
Myrcia mollis	0,16	15,97	0,55
Myrcia paivae	0,13	15,76	0,66
Myrcia splendens	0,10	21,06	0,59
Myrcia telephylla	0,15	8,02	0,77

Myrcia tenuifolia	0,13	13,62	0,86
Myrciaria floribunda	0,15	12,80	1,22
Myroxylon balsamum	0,10	26,51	0,61
Naucleopsis concinna	0,22	11,21	0,47
Naucleopsis glabra	0,16	14,34	0,57
Naucleopsis imitans	0,23	8,65	0,63
Naucleopsis krukovii	0,15	16,11	0,48
Naucleopsis macrophylla	0,14	10,80	0,45
Naucleopsis ternstroemiiflora	0,16	12,57	0,58
Naucleopsis ulei	0,18	9,23	0,57
Nealchornea yapurensis	0,17	12,76	0,51
Nectandra cuneatocordata	0,12	11,24	0,59
Nectandra globosa	0,14	11,64	0,48
Nectandra hihua	0,16	8,01	0,44
Nectandra longifolia	0,22	8,30	0,50
Nectandra membranacea	0,15	10,19	0,50
Nectandra pearcei	0,22	8,27	0,44
Nectandra pseudocotea	0,14	19,94	0,45
Nectandra reflexa	0,13	6,71	0,51
Nectandra sordida	0,19	9,58	0,39
Nectandra viburnoides	0,26	6,69	0,52
Neea divaricata	0,09	38,62	0,29
Neea floribunda	0,16	16,51	0,50
Neea laxa	0,16	22,00	0,52
Neea longipedunculata	0,15	19,02	0,53
Neea macrophylla	0,31	24,25	0,52
Neea ovalifolia	0,12	25,61	0,51
Neea parvifolia	0,27	19,10	0,40
Neea spruceana	0,22	16,89	0,48
Neea verticillata	0,27	10,78	0,37
Neea virens	0,19	18,71	0,54
Neoptychocarpus killipii	0,26	12,29	0,63
Ocotea aciphylla	0,13	12,20	0,55
Ocotea argyrophylla	0,13	9,05	0,54
Ocotea bofo	0,13	12,84	0,53
Ocotea cernua	0,13	17,30	0,47
Ocotea gracilis	0,11	16,07	0,52
Ocotea longifolia	0,18	8,62	0,42
Ocotea oblonga	0,21	12,03	0,45
Ocotea oblonga subsp. cuprea	0,13	11,80	0,44
Ocotea tabacifolia	0,11	15,58	0,40
Odontadenia funigera	0,14	14,73	0,37
Odontadenia laxiflora	0,15	33,83	0,23
Odontadenia puncticulosa	0,15	13,63	0,34
Odontadenia verrucosa	0,15	26,39	0,38
Omphalea diandra	0,18	13,17	0,28
Onychopetalum periquino	0,14	11,90	0,46
Ophiocaryon heterophyllum	0,14	9,48	0,45
Ophiocaryon klugii	0,16	6,06	0,54
Ophiocaryon manausense	0,15	30,03	0,53
Oreopanax capitatus	0,25	11,12	0,25
Ormosia amazonica	0,09	10,61	0,18
Ormosia costulata	0,09	10,05	0,41
Ormosia grandiflora	0,13	16,02	0,56
Osteophloeum platyspermum	0,20	16,33	0,43
Otoba glycycarpa	0,22	14,50	0,41
Otoba parvifolia	0,15	15,85	0,32
Ouratea pendula	0,09	18,04	0,74
Ouratea williamsii	0,13	11,04	0,78
Oxandra euneura	0,15	13,57	0,71
Oxandra mediocris	0,13	18,89	0,60
Oxandra polyantha	0,15	16,60	0,64
Oxandra xylopioides	0,18	12,43	0,63
Pachira aquatica	0,07	52,46	0,44
Pachira insignis	0,18	9,68	0,45
Pachira paraensis	0,12	16,82	0,38
Palicourea berteroana	0,12	26,13	0,43
Palicourea corymbifera	0,09	15,78	1,10
Palicourea guianensis	0,10	19,10	0,38
Palicourea hypochlorina	0,25	16,02	0,39
Palicourea longicuspis	0,14	20,52	0,57
Palicourea mansoana	0,07	18,40	0,30
Palicourea nigricans	0,10	18,42	0,41
Palicourea schunkei	0,15	26,66	0,47
Palicourea vellerea	0,15	15,94	0,44

Parinari klugii	0,13	15,08	0,59
Parinari occidentalis	0,08	18,45	0,60
Parinari parilis	0,09	19,42	0,63
Parkia nitida	0,08	12,65	0,28
Parkia panurensis	0,09	12,16	0,47
Passiflora nitida	0,12	14,05	0,39
Passiflora riparia	0,08	18,05	0,35
Paullinia bracteosa	0,10	10,33	0,33
Paullinia ingifolia	0,13	9,96	0,40
Pausandra hirsuta	0,09	16,50	0,55
Pausandra martini	0,04	14,90	0,46
Paypayrola grandiflora	0,19	14,52	0,50
Pentagonia amazonica	0,23	13,31	0,41
Pentagonia spathicalyx	0,18	10,41	0,20
Pera bicolor	0,15	14,15	0,52
Perebea angustifolia	0,11	18,55	0,69
Perebea guianensis	0,13	13,26	0,32
Perebea rubra	0,11	17,43	0,43
Peritassa huanucana	0,12	13,38	0,64
Petrea bracteata	0,37	8,26	0,48
Petrea maynensis	0,09	12,03	0,42
Phyllanthus attenuatus	0,06	23,83	0,44
Picramnia latifolia	0,11	19,50	0,51
Picramnia sellowii	0,15	12,82	0,51
Piper arboreum	0,17	24,05	0,31
Piper augustum	0,14	22,27	0,35
Piper crassinervium	0,16	27,50	0,44
Piper dumosum	0,15	24,17	0,23
Piper hispidum	0,07	22,82	0,32
Piper obliquum	0,15	18,96	0,35
Platymiscium stipulare	0,07	20,62	0,48
Pleurothyrium bifidum	0,17	8,27	0,42
Pleurothyrium insigne	0,16	9,43	0,44
Pleurothyrium panurense	0,20	8,51	0,33
Plinia duplipilosa	0,13	11,16	0,81
Plukenetia polyadenia	0,11	29,87	0,34
Posoqueria coriacea	0,19	16,12	0,59
Posoqueria latifolia	0,16	14,87	0,43
Posoqueria maxima	0,26	15,14	0,34
Potalia resinifera	0,27	8,26	0,50
Pourouma bicolor	0,15	18,98	0,42
Pourouma cecropiifolia	0,14	11,49	0,19
Pourouma cucura	0,15	10,83	0,30
Pourouma cuspidata	0,14	8,61	0,37
Pourouma guianensis	0,08	17,70	0,28
Pourouma minor	0,14	15,48	0,49
Pourouma mollis	0,16	11,03	0,30
Pourouma tomentosa	0,13	10,20	0,21
Pourouma villosa	0,20	8,37	0,28
Pouteria aubrevillei	0,16	11,11	0,61
Pouteria baehniana	0,16	10,27	0,43
Pouteria bangii	0,14	10,07	0,73
Pouteria bilocularis	0,14	12,72	0,75
Pouteria caimito	0,13	13,16	0,73
Pouteria campanulata	0,21	8,65	0,71
Pouteria cuspidata	0,18	8,94	0,64
Pouteria cuspidata subsp. robusta	0,12	17,81	0,75
Pouteria durlandii	0,17	14,72	0,53
Pouteria elegans	0,23	9,86	0,80
Pouteria ephedrantha	0,08	14,02	0,60
Pouteria filipes	0,08	19,35	0,75
Pouteria glomerata	0,13	14,52	0,67
Pouteria guianensis	0,13	12,45	0,40
Pouteria laevigata	0,21	5,35	0,52
Pouteria lucuma	0,23	9,43	0,78
Pouteria lucumifolia	0,21	11,28	0,79
Pouteria nudipetala	0,08	16,52	0,72
Pouteria oblanceolata	0,25	6,33	0,62
Pouteria opposita	0,17	7,87	0,59
Pouteria petiolata	0,14	9,49	0,68
Pouteria platyphylla	0,14	9,54	0,72
Pouteria procera	0,20	7,16	0,80
Pouteria ramiflora	0,12	9,55	0,70
Pouteria reticulata	0,17	8,14	0,59
Pouteria rostrata	0,11	6,45	0,62

Pouteria torta	0,12	12,06	0,67
Pouteria torta subsp. glabra	0,13	12,00	0,62
Pouteria torta subsp. tuberculata	0,12	7,86	0,60
Pouteria trilocularis	0,19	8,42	0,54
Pouteria vernicosa	0,11	13,47	0,58
Protium altsonii	0,10	11,99	0,52
Protium apiculatum	0,10	10,12	0,21
Protium aracouchini	0,08	18,02	0,62
Protium calendulinum	0,06	10,24	0,34
Protium carnosum	0,18	8,15	0,50
Protium crassipetalum	0,10	15,85	0,48
Protium decandrum	0,10	10,58	0,55
Protium divaricatum	0,13	12,73	0,58
Protium gallosum	0,07	10,37	0,47
Protium glabrescens	0,16	10,37	0,45
Protium hebetatum	0,11	15,08	0,58
Protium nodulosum	0,13	11,74	0,40
Protium opacum	0,13	10,91	0,48
Protium pilosissimum	0,07	14,15	0,47
Protium puncticulatum	0,06	18,17	0,37
Protium sagotianum	0,10	13,68	0,35
Protium tenuifolium	0,15	8,87	0,47
Protium trifoliolatum	0,11	14,60	0,47
Protium unifoliolatum	0,08	11,88	0,56
Prunus detrita	0,18	11,38	0,57
Pseudoconnarus macrophyllus	0,06	13,93	0,52
Pseudolmedia laevigata	0,15	15,05	0,58
Pseudolmedia laevis	0,12	16,20	0,61
Pseudolmedia macrophylla	0,12	12,57	0,57
Pseudolmedia rigida	0,15	11,91	0,54
Pseudoxandra cauliflora	0,21	13,00	0,49
Pseudoxandra lucida	0,21	13,31	0,59
Pseudoxandra pilosa	0,08	24,51	0,50
Psidium acutangulum	0,12	12,58	0,68
Psychotria bertieroides	0,13	19,40	0,45
Psychotria carthagenensis	0,18	17,78	0,52
Psychotria fortuita	0,15	18,96	0,59
Psychotria hospitalis	0,16	19,63	0,57
Psychotria remota	0,16	16,31	0,46
Psychotria zepelaciana	0,16	19,78	0,36
Pterocarpus amazonum	0,12	17,37	0,68
Pterocarpus rohrii	0,08	24,50	0,41
Pterygota amazonica	0,09	22,28	0,43
Qualea acuminata	0,06	18,40	0,50
Qualea paraensis	0,16	10,08	0,47
Qualea psidiifolia	0,09	32,52	0,58
Qualea tessmannii	0,06	40,71	0,55
Quararibea guianensis	0,14	23,20	0,52
Quiina amazonica	0,08	18,41	0,74
Quiina blackii	0,06	14,65	0,79
Quiina cruegeriana	0,11	11,71	0,74
Quiina florida	0,10	13,89	0,72
Quiina obovata	0,15	7,25	0,46
Quiina obovata	0,17	5,48	0,68
Randia armata	0,10	15,31	0,72
Raputia simulans	0,13	14,22	0,66
Rhigospira quadrangularis	0,13	11,07	0,51
Rhodostemonodaphne juruensis	0,12	15,90	0,47
Rhodostemonodaphne kunthiana	0,13	11,42	0,37
Rhodostemonodaphne sordida	0,11	14,64	0,48
Richeria grandis	0,19	11,94	0,53
Rinorea flavescens	0,11	18,94	0,69
Rinorea lindeniana	0,12	17,79	0,61
Rinorea pubiflora	0,10	26,91	0,60
Rinorea viridifolia	0,09	26,72	0,58
Roucheria columbiana	0,14	14,41	0,58
Roupala montana	0,12	8,98	0,54
Rourea accrescens	0,13	8,15	0,58
Rourea amazonica	0,10	14,17	0,56
Rourea camptoneura	0,11	10,95	0,47
Rourea cuspidata	0,11	20,38	0,62
Rudgea verticillata	0,19	18,87	0,37
Ruizterania trichanthera	0,18	9,60	0,54
Ryania speciosa	0,13	22,86	0,59
Sacoglottis ceratocarpa	0,13	10,42	0,74

Sacoglottis guianensis	0,14	13,92	0,65
Salacia caloneura	0,13	12,23	0,64
Salacia cordata	0,16	21,16	0,73
Salacia impressifolia	0,21	8,88	0,57
Salacia macrantha	0,17	10,61	0,61
Salacia ulei	0,15	11,04	0,53
Sapium marmieri	0,16	25,35	0,23
Sarcaulus brasiliensis	0,13	15,62	0,65
Sarcaulus vestitus	0,11	36,44	0,50
Schefflera morototoni	0,15	8,45	0,10
Schizocalyx peruvianus	0,14	20,61	0,37
Schnella reflexa	0,05	27,69	0,34
Schoenobiblus peruvianus	0,19	20,67	0,41
Sciadotenia toxifera	0,19	10,85	0,47
Scleronema praecox	0,16	9,11	0,66
Senefeldera inclinata	0,11	14,98	0,63
Senna macrophylla	0,05	28,68	0,25
Senna quinquangulata	0,02	58,48	0,24
Senna silvestris	0,05	29,16	0,31
Serjania elongata	0,10	14,72	0,32
Simaba guianensis	0,21	13,74	0,60
Simaba orinocensis	0,27	11,82	0,54
Simaba polyphylla	0,10	16,87	0,56
Simarouba amara	0,15	14,39	0,47
Siparuna aspera	0,12	16,25	0,21
Siparuna bifida	0,13	22,46	0,54
Siparuna cristata	0,16	23,60	0,44
Siparuna cuspidata	0,15	24,32	0,64
Siparuna decipiens	0,18	16,69	0,55
Siparuna ficoides	0,14	16,65	0,39
Siparuna guianensis	0,16	19,37	0,49
Siparuna pachyantha	0,18	15,03	0,35
Siparuna thecaphora	0,09	17,02	0,30
Sloanea eichleri	0,15	12,32	0,53
Sloanea floribunda	0,17	7,12	0,56
Sloanea fragrans	0,21	11,72	0,61
Sloanea gladysiae	0,13	11,51	0,61
Sloanea gracilis	0,10	14,70	0,72
Sloanea grandiflora	0,14	14,53	0,52
Sloanea guianensis	0,10	16,11	0,57
Sloanea latifolia	0,11	21,46	0,60
Sloanea laurifolia	0,14	6,47	0,67
Sloanea laxiflora	0,18	8,40	0,59
Sloanea macrophylla	0,10	16,81	0,48
Sloanea meianthera	0,07	26,71	0,53
Sloanea pubescens	0,10	16,86	0,57
Sloanea robusta	0,10	13,16	0,54
Sloanea rufa	0,12	12,36	0,48
Sloanea sinemariensis	0,13	6,39	0,46
Sloanea terniflora	0,09	18,12	0,64
Sloanea terniflora	0,06	21,43	0,58
Solanum barbeyanum	0,17	14,92	0,43
Solanum lepidotum	0,11	25,00	0,35
Solanum pensile	0,11	17,77	0,33
Solanum thelopodium	0,08	27,65	0,40
Solanum verecundum	0,11	21,22	0,37
Sorocea briquetii	0,11	16,93	0,56
Sorocea guilleminiana	0,19	11,85	0,56
Sorocea muriculata	0,07	24,11	0,61
Sorocea pubivena	0,10	16,64	0,54
Sorocea steinbachii	0,09	19,82	0,59
Sterculia apeibophylla	0,07	6,16	0,28
Sterculia frondosa	0,18	7,77	0,49
Sterculia pendula	0,11	14,58	0,40
Sterculia peruviana	0,12	11,78	0,27
Sterculia rebeccae	0,14	12,24	0,32
Sterculia stipulifera	0,18	9,34	0,38
Sterculia tessmannii	0,11	10,61	0,34
Sterigmapetalum obovatum	0,16	11,48	0,69
Strychnos amazonica	0,12	14,39	0,61
Strychnos bredemeyeri	0,11	25,29	0,70
Strychnos erichsonii	0,16	13,62	0,55
Strychnos jobertiana	0,14	9,49	0,57
Strychnos mitscherlichii	0,12	16,38	0,60
Strychnos sandwithiana	0,09	15,15	0,47

Stryphnodendron paniculatum	0,14	15,52	0,46
Stylogyne ardisioides	0,29	12,37	0,49
Stylogyne longifolia	0,21	12,75	0,57
Swartzia arborescens	0,10	18,77	0,72
Swartzia calva	0,19	11,00	0,50
Swartzia klugii	0,17	12,28	0,72
Swartzia myrtifolia	0,08	22,32	0,66
Swartzia oraria	0,11	15,93	0,67
Swartzia pendula	0,08	13,78	0,74
Swartzia polyphylla	0,10	17,19	0,55
Swartzia simplex	0,13	16,32	0,71
Symphonia globulifera	0,15	13,43	0,66
Tabernaemontana coriacea	0,17	13,07	0,57
Tabernaemontana flavicans	0,12	21,22	0,54
Tabernaemontana macrocalyx	0,11	13,14	0,44
Tabernaemontana sananho	0,13	22,25	0,45
Tachigali formicarum	0,08	16,83	0,40
Tachigali inconspicua	0,09	11,33	0,57
Tachigali loretensis	0,15	13,76	0,49
Tachigali pilosa	0,13	7,59	0,54
Tachigali schultesiana	0,07	15,06	0,50
Tachigali vasquezii	0,16	7,15	0,49
Talisia acutifolia	0,12	10,25	0,73
Talisia croatii	0,12	7,02	0,30
Talisia cupularis	0,10	8,94	0,78
Talisia sylvatica	0,15	6,06	0,74
Tanaecium pyramidatum	0,10	20,48	0,42
Tanaecium tetragonolobum	0,10	23,68	0,30
Tapirira guianensis	0,10	12,60	0,41
Tapirira obtusa	0,11	12,70	0,41
Tapirira retusa	0,13	8,93	0,38
Tapura amazonica	0,16	11,54	0,71
Tapura coriacea	0,20	13,18	0,67
Tapura guianensis	0,17	14,53	0,60
Tapura julianii	0,23	13,68	0,61
Tapura juruana	0,18	14,65	0,64
Telitoxicum krukovii	0,21	8,09	0,59
Telitoxicum minutiflorum	0,13	15,23	0,43
Terminalia amazonia	0,12	13,27	0,66
Terminalia chebula	0,11	16,78	0,60
Terminalia microphylla	0,08	24,30	0,55
Terminalia viridiflora	0,12	17,83	0,59
Tessmannianthus heterostemon	0,15	15,66	0,41
Tetracera volubilis	0,11	17,70	0,34
Tetragastris altissima	0,11	7,38	0,56
Tetragastris panamensis	0,12	9,34	0,57
Tetrameranthus laomae	0,12	20,84	0,63
Tetrapterys acapulcensis	0,12	18,80	0,54
Tetrorchidium macrophyllum	0,15	19,51	0,34
Theobroma cacao	0,08	19,20	0,36
Theobroma obovatum	0,10	14,95	0,55
Theobroma speciosum	0,09	7,10	0,36
Theobroma subincanum	0,16	12,47	0,39
Thyrsodium bolivianum	0,10	11,12	0,22
Thyrsodium spruceanum	0,10	11,01	0,31
Tococa macrophysca	0,07	17,63	0,53
Tococa macrosperma	0,08	17,88	0,80
Tocoyena williamsii	0,09	29,49	0,36
Tontelea attenuata	0,12	15,90	0,71
Tontelea mauritioides	0,17	15,08	0,51
Tontelea passiflora	0,22	9,20	0,66
Toulicia reticulata	0,11	10,32	0,37
Tovomita auriculata	0,20	6,86	0,64
Tovomita carinata	0,23	10,71	0,75
Tovomita krukovii	0,15	12,62	0,69
Tovomita longifolia	0,34	6,50	0,49
Tovomita stylosa	0,14	21,03	0,75
Trattinnickia aspera	0,07	18,75	0,24
Trattinnickia boliviana	0,10	14,69	0,31
Trema micrantha	0,09	46,59	0,25
Trichilia elegans	0,06	28,41	0,58
Trichilia micrantha	0,16	20,51	0,49
Trichilia microspadyx	0,12	10,20	0,67
Trichilia pallida	0,12	16,81	0,66
Trichilia pittieri	0,10	8,89	0,45

Trichilia pleeana	0,14	12,40	0,71
Trichilia poeppigii	0,17	20,60	0,66
Trichilia quadrijuga	0,10	11,17	0,42
Trichilia rubra	0,16	7,55	0,60
Trichilia septentrionalis	0,12	12,06	0,41
Trichilia singularis	0,10	18,80	0,79
Trichilia solitudinis	0,16	8,65	0,51
Trichilia tuberculata	0,13	14,30	0,74
Trigynaea duckei	0,14	19,43	0,53
Triplaris americana	0,10	18,35	0,24
Triplaris poeppigiana	0,10	27,80	0,40
Trischidium alternum	0,11	24,57	0,64
Trymatococcus amazonicus	0,08	17,99	0,61
Turpinia occidentalis	0,13	21,20	0,29
Tynanthus panurensis	0,10	21,54	0,39
Unonopsis floribunda	0,11	16,71	0,37
Unonopsis spectabilis	0,12	13,07	0,46
Vantanea guianensis	0,15	11,29	0,82
Vatairea erythrocarpa	0,13	10,60	0,44
Vatairea fusca	0,05	22,43	0,32
Virola calophylla	0,14	11,82	0,61
Virola decorticans	0,11	18,75	0,39
Virola duckei	0,11	14,89	0,33
Virola elongata	0,10	16,43	0,47
Virola flexuosa	0,09	31,39	0,33
Virola multicostata	0,11	16,03	0,41
Virola multinervia	0,13	11,42	0,36
Virola pavonis	0,15	13,54	0,45
Virola sebifera	0,14	17,63	0,42
Vismia glabra	0,15	19,39	0,47
Vitex triflora	0,06	36,38	0,56
Vochysia venulosa	0,19	13,70	0,65
Vochysia vismiifolia	0,16	12,56	0,52
Warszewiczia coccinea	0,12	18,46	0,64
Warszewiczia cordata	0,12	17,88	0,54
Warszewiczia elata	0,14	17,35	0,64
Xylopia calophylla	0,11	17,60	0,51
Xylopia cuspidata	0,14	16,80	0,69
Xylopia excellens	0,11	14,62	0,60
Xylopia nitida	0,19	12,17	0,28
Zanthoxylum acuminatum	0,16	10,53	0,42
Zanthoxylum huberi	0,14	21,57	0,79
Zanthoxylum rhoifolium	0,06	1,52	0,48
Zanthoxylum riedelianum	0,12	19,58	0,31
Zygia coccinea	0,14	14,27	0,61
Zygia macbridei	0,17	10,83	0,72
